# Azithromycin Resistance in Salmonella Typhi and Paratyphi Across South Asia: A Systematic Review and Meta-Analysis

**DOI:** 10.1093/ofid/ofag581

**Published:** 2026-09-12

**Authors:** Aftab Ullah, Asif Jan, Muhammad Shabil, Haiqa Sayyed, Muhammad Mustafa Khan, Muhammad Akhlaq, Abdur Rahim, Daniel Dubwa

**Affiliations:** Department of Pharmacy, Hazara University, Mansehra, KP, Pakistan; District Headquarters Hospital, Charsadda, KP, Pakistan; Saidu Group of Teaching Hospital, Saidu Sharif, Swat, KP, Pakistan; Department of Pharmacy, University of Peshawar, Peshawar, KP, Pakistan; Faculty of Pharmacy, MS Ramaiah University of Applied Sciences, Bangalore, India; Khyber Medical College, Peshawar, KP, Pakistan; Khyber Medical College, Peshawar, KP, Pakistan; Department of Pharmacy, Hazara University, Mansehra, KP, Pakistan; CECOS University of IT and Emerging Sciences, Peshawar, KP, Pakistan; Department of Pharmacy, Makerere University, Kampala, Uganda

**Keywords:** antimicrobial resistance, azithromycin resistance, Salmonella Paratyphi, Salmonella Typhi, South Asia

## Abstract

Azithromycin is a frontline oral treatment for uncomplicated enteric fever in South Asia; however, emerging resistance in *Salmonella enterica* serovars Typhi and Paratyphi challenges its long-term efficacy. We conducted a PRISMA-compliant systematic review and meta-analysis, searching 6 databases to estimate the prevalence of azithromycin resistance in low-income and developing countries in South Asia. Accepted resistance classifications were based on interpretive criteria used by individual study authors, acknowledging that the Clinical and Laboratory Standards Institute breakpoints for *S. Typhi* were introduced in 2015 and that no official breakpoints exist for *S. Paratyphi*. From 1863 screened records, 74 studies comprising 30 085 isolates were analyzed using random-effects models. The pooled azithromycin resistance was 5% (95% CI: 4%–5%) for *S. Typhi* and 5% (95% CI: 0%–9%) for *S. Paratyphi*. When both serotypes were combined, the overall prevalence was 9% (95% CI: 3%–14%). Subgroup analysis revealed significant geographical heterogeneity: Pakistan and India reported the highest prevalence (8% and 7%, respectively), while Nepal and Bangladesh remained at 1%. Meta-regression did not detect a statistically significant temporal trend from 2005 to 2024. Although overall resistance remains relatively low, high prevalence in specific regions necessitates rigorous antimicrobial stewardship and enhanced surveillance to preserve azithromycin's clinical utility against enteric fever in South Asia.

## Teaser

This meta-analysis of 74 South Asian studies involving 30,085 clinical isolates reveals a stable 5% pooled azithromycin resistance for both Salmonella Typhi and Paratyphi. Resistance patterns vary geographically, peaking at 8% in Pakistan and 7% in India.

## Graphical Abstract

**Graphical Abstract:**
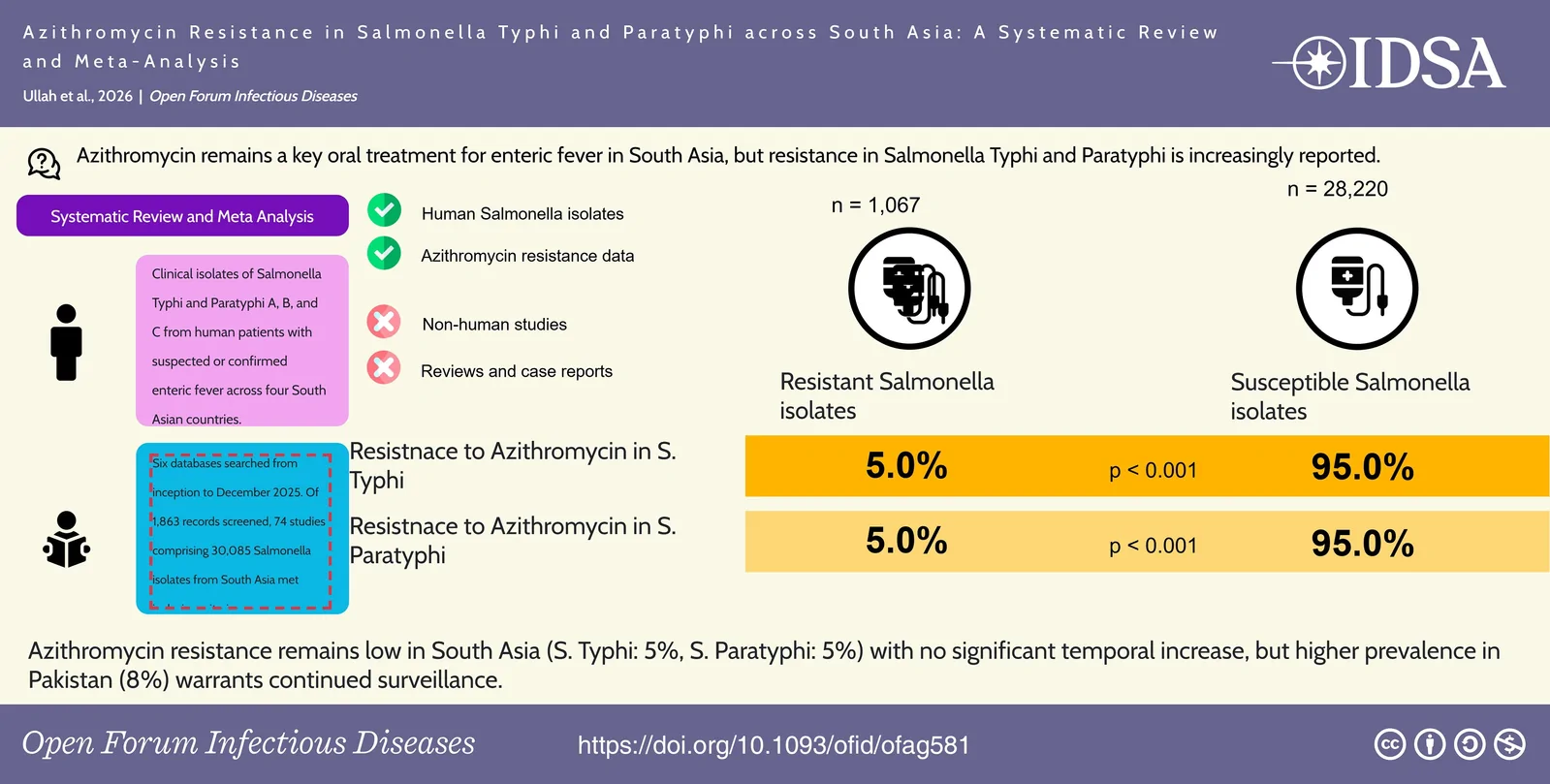

This graphical abstractis also available at Tidbit: https://tidbitapp.io/tidbits/azithromycin-resistance-in-salmonella-typhi-and-paratyphi-across-south-asia-a-systematic-review-and-meta-analysis-1270ad1e-cd06-4edb-89c4-6da155113f14

Enteric fever, caused primarily by *Salmonella enterica* serovars *Typhi* and *Paratyphi* A, B, and C, remains a major public health problem in low- and middle-income countries, particularly in South Asia [1]. The disease is transmitted through contaminated food and water and is closely associated with inadequate sanitation, limited access to clean drinking water, and high population density. Countries such as Pakistan, India, Bangladesh, and Nepal collectively account for a substantial proportion of the global burden of enteric fever, with millions of cases reported annually and significant associated morbidity and mortality [2]. Despite advances in prevention strategies, including improvements in water, sanitation, and hygiene (WASH) and the introduction of typhoid conjugate vaccines, antimicrobial therapy continues to play a central role in the management of enteric fever [3].

Historically, first-line agents such as chloramphenicol, ampicillin, and trimethoprim–sulfamethoxazole were highly effective in treating enteric fever [4]. However, the widespread emergence of multidrug-resistant (MDR) *Salmonella* strains since the late 20th century severely compromised the utility of these antibiotics [5]. Subsequently, fluoroquinolones became the treatment of choice, but their extensive use led to the rapid development of reduced susceptibility and resistance, particularly in South Asia [6]. More recently, the emergence and spread of extensively drug-resistant (XDR) *Salmonella Typhi*, especially in Pakistan, has further narrowed therapeutic options and heightened concerns regarding effective case management [7].

Azithromycin, a macrolide antibiotic with favorable oral bioavailability, intracellular penetration, and a relatively good safety profile, has emerged as a key oral treatment option for uncomplicated enteric fever. It is recommended by several national and international guidelines, particularly in settings where fluoroquinolone resistance is prevalent and third-generation cephalosporins are reserved for severe disease. Azithromycin has also been widely used in pediatric populations, where treatment options are more limited. However, increasing reports of azithromycin resistance in *Salmonella* species, including treatment failures and elevated minimum inhibitory concentrations (MICs), have raised alarms regarding the long-term effectiveness of this critical antibiotic.

A critical consideration in interpreting azithromycin resistance data is the historical evolution of antimicrobial susceptibility testing (AST) interpretive criteria. The Clinical and Laboratory Standards Institute (CLSI) first established breakpoints for azithromycin against Salmonella Typhi in January 2015 (M100-S25), defining susceptible (MIC ≤16 µg/mL) and resistant (MIC ≥32 µg/mL) categories. The European Committee on Antimicrobial Susceptibility Testing (EUCAST) subsequently adopted similar criteria. Critically, however, no official CLSI or EUCAST breakpoints have been established for Salmonella Paratyphi serotypes A, B, or C, which account for a substantial proportion of paratyphoid fever cases in South Asia. Among these, Paratyphi A is the predominant cause of paratyphoid fever in the region, while serotypes B and C are rarely encountered. This regulatory gap has necessitated the use of *S. Typhi* breakpoints for *S. Paratyphi* isolates in published studies, despite evidence suggesting that epidemiological MIC distributions may differ between serotypes. Consequently, prevalence estimates for *S. Paratyphi* derived from these studies, and particularly combined serotype analyses, warrant careful methodological scrutiny.

South Asia represents a unique epidemiological setting for the emergence and dissemination of antimicrobial resistance in enteric fever pathogens due to high disease incidence, widespread empirical antibiotic use, over-the-counter availability of antimicrobials, and variable laboratory capacity for AST [8]. Although numerous individual studies have reported azithromycin resistance among *Salmonella* isolates across the region, the reported prevalence varies widely by country, study period, population, and laboratory methodology. This heterogeneity makes it difficult to draw robust conclusions about the true burden and trends in azithromycin resistance, limiting clinicians' and policymakers' ability to make evidence-based decisions.

Previous reviews have largely focused on overall antimicrobial resistance patterns in enteric fever or have been restricted to single countries or limited time periods [3, 9, 10]. To date, there has been no comprehensive meta-analysis synthesizing available data on azithromycin resistance in *Salmonella Typhi* and *Salmonella Paratyphi* across South Asia. A pooled regional estimate is essential for contextualizing local findings, identifying geographical variations, assessing temporal trends, and guiding treatment guidelines, surveillance priorities, and antimicrobial stewardship strategies. Therefore, the present systematic review and meta-analysis aimed to estimate the pooled prevalence of azithromycin resistance in *Salmonella* Typhi and *Salmonella* Paratyphi isolates from human populations in South Asia.

## METHODS

The study was conducted and reported in accordance with PRISMA guidelines (Supplementary Table 1) and registered with PROSPERO. Rayyan.ai (a web-based software) was used for deduplication and screening of the articles.

### Search Strategy

A comprehensive search was conducted in Embase, Google Scholar, PubMed, ScienceDirect, Web of Science, and the local database of PakMediNet to identify and retrieve potentially relevant studies from inception. The literature search was conducted on 31 December 2025. The search terms used were (“Salmonella typhi” OR “Salmonella paratyphi A” OR “Salmonella paratyphi B” OR salmonella OR “typhi” OR paratyphi OR “S. typhi” OR “S. paratyphi”) AND (“azithromycin” OR “azithromycin” OR “macrolide”) AND (“resistant” OR “antimicrobial susceptibility” OR “antibiotic susceptibility”) AND (“South Asia” OR “Pakistan” OR “India” OR “Bangladesh” OR “Nepal” OR “Bhutan” OR “Sri Lanka”) (Supplementary Table 2).

### Interpretive Criteria and Breakpoint Definitions

Interpretive criteria for azithromycin susceptibility testing varied across the included studies. CLSI established breakpoints for *S. Typhi* in 2015 (susceptible MIC ≤16 µg/mL; resistant ≥32 µg/mL), while no official breakpoints exist for *S. Paratyphi*. Studies reporting *S. Paratyphi* resistance have necessarily applied *S. Typhi* breakpoints or laboratory-derived criteria, a limitation acknowledged in our interpretation. We accepted the criteria used by individual study authors and conducted a sensitivity analysis comparing pre- and post-2015 studies to assess the impact of breakpoint standardization on resistance estimates.

### Inclusion and Exclusion Criteria

All original articles (cross-sectional, prospective, or retrospective studies) that reported the prevalence of azithromycin resistance in Salmonella isolates from human populations in South Asia were considered for the study. Case reports, conference presentations, reviews, studies not available in English, and articles with insufficient methodological detail to determine resistance prevalence (eg, studies reporting only qualitative susceptibility categories without specifying interpretive criteria or total isolate numbers) and studies evaluating isolates from nonhuman populations were excluded. Antibiotic susceptibility testing in nonhuman populations was also a criterion for exclusion (Supplementary Table 3).

### Screening

Rayyan.ai (a web-based systematic review platform) was used for deduplication and screening of the articles. All screening was performed independently and in duplicate. After removing irrelevant papers, 2 authors (A. U. and M. S.) independently scrutinized the titles and abstracts. In the second step, 2 authors (H. S. and A. J.) independently evaluated the entire texts of the articles. Discrepancies regarding the inclusion of studies between the reviewers were resolved by consulting a third reviewer (M. A.).

### Data Extraction

Two researchers (A. U. and M. K.) independently extracted data from potentially relevant studies, including the first author, publication year, study design, sample size, patient demographics (age and sex), type of diagnostic test used for antibiotic susceptibility, prevalence of resistance to specific antibiotics, country, and year(s) of data collection. All potential discrepancies among the reviewers were resolved through discussion to reach a consensus.

### Quality Assessment

Methodological quality was assessed using the Joanna Briggs Institute (JBI) Critical Appraisal Checklist for Prevalence Studies, with each of 9 domains scored as “Yes,” “No,” or “Unclear.” For AST validity (domains 6 and 7), we evaluated adherence to CLSI/EUCAST guidelines, use of quality control strains, clear description of testing methodology, and evidence of external quality assurance. Studies satisfying all 4 criteria were classified as high quality for AST domains; those with insufficient methodological detail were classified as low quality. Overall quality (moderate or low) was determined by the proportion of criteria satisfied across all domains. Each article was independently assessed by 2 reviewers, with disagreements resolved through consensus (Supplementary Table 4).

### Statistical Analysis

All statistical analyses were performed using R software (Version 4.5.1) with the “meta” and “metafor” packages. Random-effects meta-analyses were conducted using the inverse-variance method to pool resistance proportions, separately for *S. Typhi, S. Paratyphi*, and for combined serotypes. The Freeman–Tukey double arcsine transformation was applied to stabilize variances, with pooled estimates back-transformed to percentages for presentation. Subgroup analyses were performed by country (Pakistan, India, Nepal, and Bangladesh) to explore geographical heterogeneity. Statistical heterogeneity was quantified using the *I*^2^ statistic, with values of 25%, 50%, and 75% considered indicative of low, moderate, and high heterogeneity, respectively. To evaluate temporal trends, univariate meta-regression was performed using the midpoint year of sample collection as a continuous predictor variable in the logit-transformed proportion model. Publication bias was assessed through visual inspection of funnel plots and, where appropriate, Egger's test for small-study effects.

### Sensitivity Analysis

To assess the potential impact of the 2015 CLSI breakpoint introduction on resistance estimates, we conducted a subgroup sensitivity analysis comparing studies conducted before and after January 2015. This analysis was performed separately for *S. Typhi* and *S. Paratyphi* and is presented in Supplementary Table 5. The presence of studies that used different interpretive criteria before 2015 is acknowledged as a potential source of heterogeneity.

## RESULTS

### Literature Search and Study Selection

**Figure 1.**
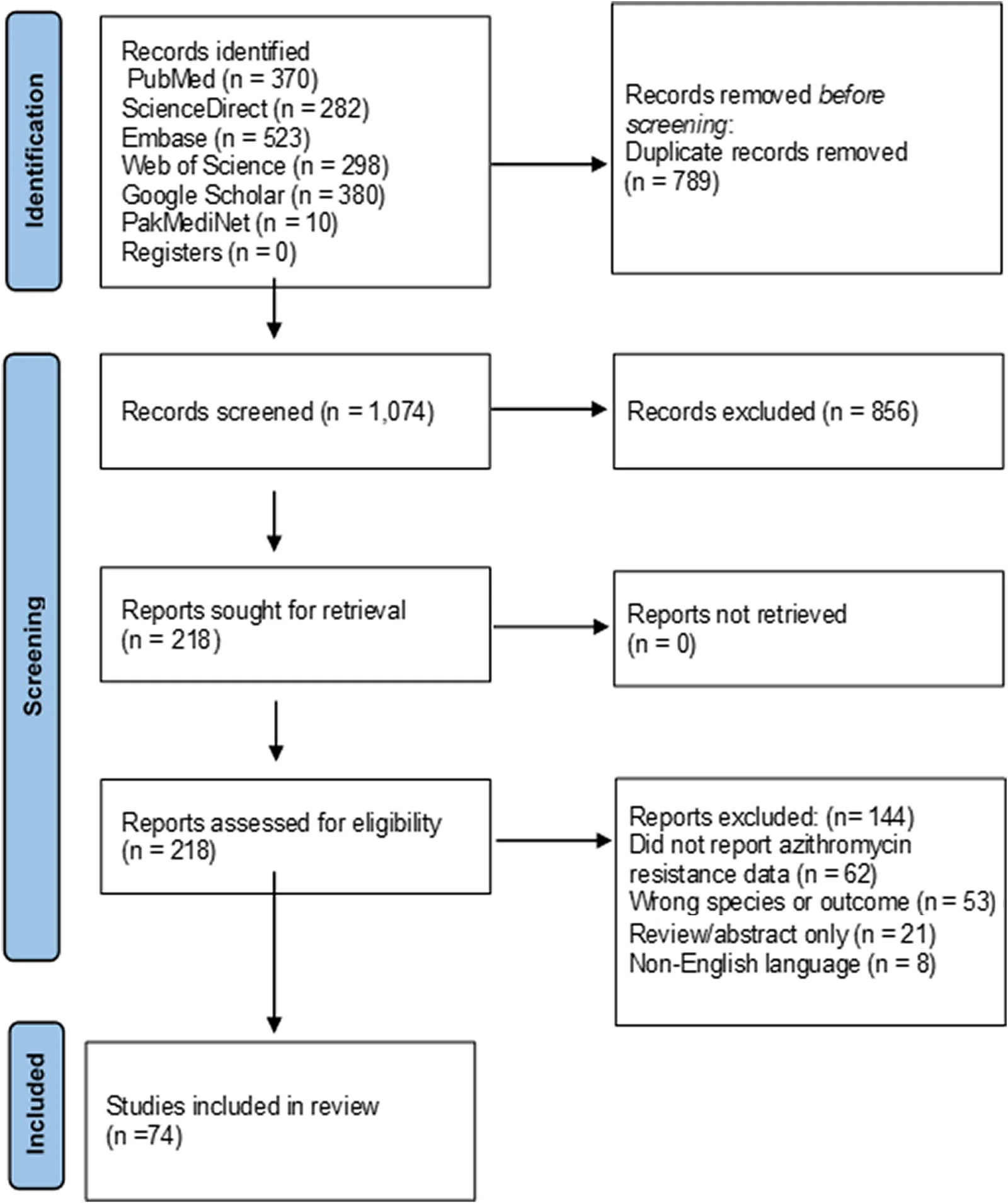
PRISMA flow diagram of study selection.

A comprehensive literature search across 6 databases (PubMed, ScienceDirect, Embase, Web of Science, Google Scholar, and PakMediNet) yielded 1863 records. After removing 789 duplicate entries, 1074 unique records underwent title and abstract screening. Of these, 218 studies were deemed relevant for full-text review. Following a detailed evaluation, 144 studies were excluded for the following reasons: 62 did not report the prevalence of azithromycin resistance; 53 investigated non-Salmonella species or different outcomes; 21 were review articles or conference abstracts; and 8 were not available in English. Consequently, 74 studies met all eligibility criteria and were included in the final meta-analysis. The study selection process is detailed in the PRISMA flow diagram (Figure 1).

The majority of the included studies were from Pakistan [11–44], followed by India [45–70], Nepal [71–77], and Bangladesh [78–82]. One multicountry surveillance study was also included [83]. Although Sri Lanka and Bhutan were included in the search strategy, no eligible studies from these countries were found to meet the inclusion criteria. Study designs were primarily cross-sectional, retrospective, or laboratory-based surveillance. Detailed characteristics of each study, including country, setting, design, sample size, specimen type, population, study period, and AST method, are summarized in Table 1.

**Table 1.** Characteristics of Included Studies

Study ID	Country	Study Setting	Study Design	Sample Size	Specimen	Population	Study Period	AST Method	Serotype Reported
Ahsan 2019 [78]	Bangladesh	Dhaka, local hospital	Cross-sectional	40.0	Feces	-	June 2015 and 2016	Disc diffusion, MIC by broth dilution	*S. Typhi*, *S. Paratyphi*
Shah 2020 [11]	Pakistan	Wahcantt hospital	Cross-sectional	81.0	Blood	Culture-positive typhoid patients >12 y	Jan-Sep 2019	Kirby–Bauer disc diffusion	*S. Typhi*
Aziz 2018 [12]	Pakistan	Pediatric unit of Karachi	Cross-sectional	137.0	Blood	Children 1.6–11 y with enteric fever	June-Sep 2018	Kirby–Bauer disc diffusion	*S. Typhi*
Behl 2017 [45]	India	Chandigarh	Retrospective, laboratory-based observational	106.0	Blood, pus, stool	Culture-confirmed salmonellosis	Jan 2011-June 2012	Kirby–Bauer, MIC by agar dilution/E-test	*S. Typhi*, *S. Paratyphi* A, S. Typhimurium, S. Enteritidis
Bhetwal 2017 [71]	Nepal	Kathmandu	Laboratory-based descriptive	2304.0	Blood	Suspected enteric fever	Apr 2015-Mar 2017	Kirby–Bauer disc diffusion	*S. Typhi*
Butt 2011 [13]	Pakistan	Shaukat Khanam hospital	Laboratory-based antimicrobial susceptibility	45.0	Blood	Suspected enteric fever	Nov 2007-June 2009	Disc diffusion	*S. Typhi*, *S. Paratyphi* A, B, C
Choudhary 2013 [46]	India	Chennai	Observational laboratory-based	322.0	Blood	-	May 2009-June 2011	Kirby–Bauer disc diffusion	*S. Typhi*, *S. Paratyphi* A, B
Harichandran 2017 [47]	India	Kerala	Observational laboratory-based descriptive	118.0	Blood, sterile fluids, stool, urine	-	Aug 2011-July 2013	Kirby–Bauer disc diffusion	*S. Typhi*, *S. Paratyphi* A, S. Typhimurium
Hooda 2019 [79]	Bangladesh	2 pediatric hospitals	Surveillance	1082.0	Blood	-	2009–2016	Disc diffusion, E-test	*S. Typhi*, *S. Paratyphi* A
Jabeen 2023 [14]	Pakistan	Lahore	Retrospective cross-sectional validation	835.0	Blood	-	Jan-June 2021	Kirby–Bauer disc diffusion	*S. Typhi*
Jain 2013 [48]	India	New Delhi	Observational laboratory	344.0	Blood	Enteric fever patients	Jan 2010-July 2012	Kirby–Bauer/Phoenix 100	*S. Typhi*, Paratyphi A, B
Rasheed 2023 [15]	Pakistan	TCH Lahore	Descriptive	1306.0	Blood	Pediatric patients 0–12 y	Jan 2021-Dec 2022	VITEK 2 Compact	*S. Typhi*
Shaikh 2019 [16]	Pakistan	Sukkar	Retrospective	465.0	Blood	-	Jan-Dec 2017	Kirby–Bauer disc diffusion	*S. Typhi*, Paratyphi A, B
Shah 2024 [17]	Pakistan	Peshawar	Prospective observational cohort	530.0	Blood, feces, urine	Blood culture-positive patients	2 y	Broth microdilution/disk diffusion	*S. Typhi*, *S. Paratyphi*
Ahmad 2020 [84]	Pakistan	Peshawar	Descriptive	168.0	Blood	Children with positive blood culture	June 2019-May 2020	Kirby–Bauer disc diffusion	*S. Typhi*, *S. Paratyphi* A
Batool 2021 [19]	Pakistan	Lahore	Retrospective	2575.0	Blood	Pediatric sepsis patients	Sep 2018-Feb 2019	VITEK 2 Compact	*S. Typhi*, *S. Paratyphi* A
khan 2022 [20]	Pakistan	Karachi	Retrospective	120.0	Blood	Patients 5–15 y	June-Dec 2020	Kirby–Bauer disc diffusion	*S. Typhi*, *S. Paratyphi*
Khan 2023 [21]	Pakistan	Peshawar	Cross-sectional	100.0	Blood	Patients 15–25 y	Jan-Dec 2019	Kirby–Bauer disc diffusion	Paratyphi, Typhi
Aslam 2021 [22]	Pakistan	Lahore	Descriptive cross-sectional	1510.0	Blood	Culture-positive patients	Jan 2019-Mar 2020	Kirby–Bauer disc diffusion	*S. Typhi*, *S. Paratyphi*
Baig 2023 [23]	Pakistan	Lahore	Cross-sectional	105.0	Blood	Patients 0–15 y	July 2020-Jan 2021	Kirby–Bauer disc diffusion	*S. Typhi*, *S. Paratyphi*
Laghari 2019 [24]	Pakistan	Jamshoro	Cross-sectional	275.0	Blood	Children ≤18 y	July-Dec 2018	Kirby–Bauer disc diffusion	*S. Typhi*, *S. Paratyphi* A, B
Fida 2021 [25]	Pakistan	CMH Lahore	Cross-sectional observational	52.0	Blood	Culture-positive patients	Apr-Oct 2019	NR	*S. Typhi*, Paratyphi
Hussain 2022 [26]	Pakistan	Kharian	Cross-sectional	315.0	Blood	-	Jan 2019-Sep 2020	Kirby–Bauer disc diffusion	*S. Typhi*, *S. Paratyphi* A-C
Hussain 2019 [27]	Pakistan	Karachi	Prospective	430.0	Blood	Enteric fever patients	Jan-Dec 2018	Kirby–Bauer disc diffusion	*S. Typhi*, *S. Paratyphi* A
Joshi 2018 [49]	India	Bangalore	12-y record-based	3298.0	Blood	-	Jan 2002-Dec 2013	Kirby–Bauer disc diffusion, E-test	*S. Typhi*, *S. Paratyphi* A
Khadka, S 2021 [72]	Nepal	Kathmandu	Laboratory-based cross-sectional	706.0	Blood	Febrile outpatients	June-Sep 2018	Kirby–Bauer, agar dilution MIC	*S. Typhi*
Khadka, T 2021 [73]	Nepal	Kathmandu	Cross-sectional	1815.0	Blood	-	Apr 2017-Mar 2018	Kirby–Bauer disc diffusion	*S. Typhi*, *S. Paratyphi*
Khan 2024 [28]	Pakistan	Peshawar	Prospective cross-sectional	562.0	Blood	-	Oct-Dec 2020	Kirby–Bauer disc diffusion, MIC	*S. Typhi*, *S. Paratyphi* A
Khanal 2017 [74]	Nepal	Kathmandu	Cross-sectional	88.0	Blood	Suspected enteric fever	Mar-Aug 2016	Kirby–Bauer, agar dilution MIC	*S. Typhi*, *S. Paratyphi*
Khattak 2025 [29]	Pakistan	KPK	Cross-sectional	5640.0	Blood	Suspected enteric fever	Sep 2022-Aug 2023	Kirby–Bauer disc diffusion	*S. Typhi*
Khatun 2018 [80]	Bangladesh	Dhaka	Cross-sectional	702.0	Blood and stool	-	Jan 2010-Dec 2014	Disc diffusion	*S. Typhi*, *S. Paratyphi* A
Kokare 2021 [50]	India	Mumbai	Retrospective	155.0	Blood	-	Jan 2016-Dec 2018	Broth microdilution	*S. Typhi*, *S. Paratyphi* A
Makkar 2019 [51]	India	North India	Ambispective	29 184.0	Blood	-	Jan 2011-Dec 2017	Vitek 2 Compact	*S. Typhi*, *S. Paratyphi* A, B
Malla 2005 [75]	Nepal	Kathmandu hospitals	Prospective epidemiological	1469.0	Blood	Enteric fever patients	June 2002-July 2004	Disc diffusion, agar dilution MIC	*S. Typhi*, *S. Paratyphi* A
Misra 2015 [66]	India	Lucknow	Descriptive observational	22 876.0	Blood	Suspected enteric fever	Jan 2013-Dec 2014	Kirby–Bauer disc diffusion, E-test	*S. Typhi*, *S. Paratyphi* A
Misra 2016 [67]	India	Lucknow	Laboratory-based antimicrobial susceptibility	100.0	Blood	Culture-confirmed Salmonella	Jan 2013-Dec 2015	Kirby–Bauer disc diffusion, E-test	*S. Typhi*, *S. Paratyphi* A, B
Okanda 2018 [81]	Bangladesh	Dhaka	Laboratory-based observational	82.0	Blood	-	Aug-Oct 2015	Broth microdilution	*S. Typhi*, *S. Paratyphi* A
Patel 2017 [52]	India	Varanasi	Prospective case study	126.0	Blood, stool, urine	-	Feb 2011-Mar 2013	Kirby–Bauer disc diffusion, MIC	*S. Typhi*
Patil 2019 [68]	India	Mumbai	In vitro observational	251.0	Blood	Culture-positive patients	Apr-Aug 2018	E-test method	*S. Typhi*, *S. Paratyphi* A
Qamar 2020 [83]	Bangladesh, Nepal, Pakistan	-	Surveillance	8705.0	Blood	Blood culture-confirmed enteric fever	Sep 2016-Sep 2019	Disc diffusion	*S. Typhi*, *S. Paratyphi*
Qamar 2025 [30]	Pakistan	5 large laboratories	Retrospective review	464 956	Blood	-	Jan 2017-Dec 2019	NR	*S. Typhi*, *S. Paratyphi*
Rai 2012 [53]	India	North India TCH	Observational analytical	107.0	Blood	-	2005–2008	Disc diffusion	*S. Typhi*, *S. Paratyphi* A
Sahai 2025 [54]	India	8 sites in 7 cities	Multicenter surveillance	270 228.0	Blood and bone marrow	-	2021–2024	Kirby–Bauer, broth microdilution MIC	*S. Typhi*, *S. Paratyphi* A
Sattar 2020 [31]	Pakistan	Sialkot	Observational laboratory-based	2773.0	Blood	-	Jan-June 2019	Modified Kirby–Bauer	*S. Typhi*, *S. Paratyphi*
Sharma 2019 [55]	India	New Delhi	Retrospective lab-based molecular	602.0	Blood	Enteric fever patients	1993–2016	Disc diffusion, E-test	*S. Typhi*, *S. Paratyphi* A
Singhal 2014 [56]	India	North India	Retrospective analysis	1038.0	Blood	-	Jan 2001-Dec 2012	Disc diffusion	*S. Typhi*, *S. Paratyphi* A
Srirangaraj 2014 [57]	India	Pondicherry	Retrospective laboratory-based	16.0	Blood	-	Jan 2012-June 2013	Kirby–Bauer disc diffusion	*S. Typhi*
Umair 2020 [32]	Pakistan	Islamabad	Retrospective cross-sectional	664.0	Blood	Patients ≥12 y with positive cultures	2012–2018	NR	*S. Typhi*, *S. Paratyphi*
Tariq 2025 [33]	Pakistan	Quetta	Cross-sectional	480.0	Blood	Widal positive cases	Mar 2022–2023	Kirby–Bauer disc diffusion	*S. Typhi*
Vala 2016 [58]	India	Ahmedabad, Vadodara, Bhavnagar	Longitudinal community-based	61.0	Blood	-	Jan-June 2011	Kirby–Bauer, agar dilution MIC	*S. Typhi*
Veeraraghavan 2021 [59]	India	19 centers nationwide	Multicenter surveillance	2373.0	Blood	Blood culture-positive participants	Nov 2017-Jan 2020	Kirby–Bauer, broth microdilution MIC	*S. Typhi*, *S. Paratyphi* A
Yasin 2022 [34]	Pakistan	North-west Pakistan	Cross-sectional	2138.0	Blood	Fever/enteric fever symptoms	May 2016-Apr 2017	NR	*S. Typhi*, *S. Paratyphi* A, B
Kamaal 2018 [60]	India	Aligarh	Prospective nonrandomized	142.0	Blood	Pediatric patients ≤15 y	Sep 2005-Aug 2006	Kirby–Bauer disc diffusion	*S. Typhi*, Paratyphi A, B
Sajib 2023 [82]	Bangladesh	Dhaka	Long-term surveillance	2725.0	Blood	Hospital and OPD patients	1999–2021	Kirby–Bauer, broth microdilution MIC	*S. Paratyphi* A
Noor 2025 [35]	Pakistan	KPK	Retrospective descriptive	621.0	Blood	Urban and rural enteric fever patients	Jan 2019-Dec 2022	Disk diffusion and MIC	*S. Typhi*, *S. Paratyphi*
Afzal 2024 [36]	Pakistan	Nawabshah	Cross-sectional	300.0	Blood	Typhoid patients	Dec 2022-June 2023	Biochemical typing, disk diffusion, MIC	*S. Typhi*
Ahmed 2023 [44]	Pakistan	Peshawar	Cross-sectional	210.0	Blood	Symptomatic typhoid patients	Apr 2021-Mar 2022	Kirby–Bauer disc diffusion	*S. Typhi*
Ali 2022 [38]	Pakistan	Buner	Cross-sectional	460.0	Blood	Typhoid fever symptoms	Jan 2020-May 2021	Kirby–Bauer disc diffusion	*S. Typhi*
Iyer 2017 [61]	India	South India	Retrospective	77 713.0	Blood	-	Jan 2007-Dec 2016	BSAC/EUCAST guidelines	*S. Typhi*, *S. Paratyphi* A
Bhatia 2025 [62]	India	Northern India	Observational	18.0	Blood and pus	Patients 2–55 y	Jan 2019-Jan 2023	VITEK 2 Compact	*S. Typhi*, *S. Paratyphi*
Safi 2022 [39]	Pakistan	Peshawar	Laboratory-based observational	100.0	Blood	Suspected typhoid patients 15–50 y	Jan-Mar 2019	Disc diffusion	*S. Typhi*, *S. Paratyphi* A
Mahmood 2024 [27]	Pakistan	Peshawar	Cross-sectional	84.0	Blood	XDR *S. Typhi* infection	Mar-Sep 2022	Disc diffusion	*S. Typhi*
Chitkara 2019 [63]	India	North-West Delhi	Case record analysis	166.0	Blood	Children with suspected enteric fever	Apr 2010-Mar 2016	VITEK 2 Compact	*S. Typhi*, *S. Paratyphi* A
Hayat 2025 [30]	Pakistan	Faisalabad	Retrospective analysis	2456.0	Blood	-	NR	Disc diffusion	*S. Typhi*
Gupta 2013 [69]	India	Chandigarh	Descriptive longitudinal observational	8142, 8134, 8114	Blood	Pediatric and male patients	2008, 2009, 2010	Kirby–Bauer, agar dilution/E-test MIC	*S. Typhi*, *S. Paratyphi* A
Maqsood 2024 [41]	Pakistan	Karachi	Cross-sectional	77.0	Blood	Suspected typhoid patients	Sep 2021-May 2022	Kirby–Bauer disc diffusion	*S. Typhi*
Capoor 2007 [64]	India	New Delhi	Lab-based in vitro observational	384.0	Blood	Enteric fever patients	Dec 2004-Dec 2006	Kirby–Bauer disc diffusion	*S. Typhi*, *S. Paratyphi* A
Sharma 2018 [65]	India	New Delhi	Retrospective observational trend	329 232.0	Blood	Culture-positive enteric fever	Jan 2005-Dec 2016	Disk diffusion, E-test MIC	*S. Typhi*, *S. Paratyphi* A
Joshi 2018 [49]	Nepal	Kathmandu	Retrospective	159.0	Blood	Patients >15 y with enteric fever	Jan 2012-Dec 2016	Disc diffusion	*S. Typhi*, *S. Paratyphi*
Muhammad 2025 [43]	Pakistan	Peshawar	Observational lab-based	2950.0	Blood	Suspected typhoid patients	NR	Kirby–Bauer disc diffusion	*S. Typhi*
Chapagain 2023 [77]	Nepal	Kathmandu	Retrospective	121.0	Blood	-	Apr 2016-Mar 2022	NR	*S. Typhi*, Paratyphi A, B
Garg 2013	India	North India	Observational combined laboratory	42.0	Blood	Patients with fever	Feb 2012-Jan 2013	Kirby–Bauer disc diffusion	*S. Typhi*, *S. Paratyphi* A
Qaiser 2011 [42]	Pakistan	Karachi	-	154.0	Blood	-	Mar-Aug 2007	Kirby–Bauer disc diffusion	*S. Typhi*, *S. Paratyphi* A, B, C
Ahmad 2023 [37]	Pakistan	Lahore	Observational	246.0	Blood	Suspected enteric fever	Nov 2020-May 2021	Kirby–Bauer disc diffusion	*S. Typhi*, Paratyphi A or B

### Quality Assessment

Methodological quality was assessed using the JBI checklist. Of the 74 included studies, 56 (75.7%) were rated as moderate quality, while 18 (24.3%) were rated as low quality due to insufficient reporting of AST methodology, quality control procedures, or study setting details. The complete quality assessment is presented in Supplementary Table 4.

### Prevalence of Azithromycin Resistance

**Figure 2.**
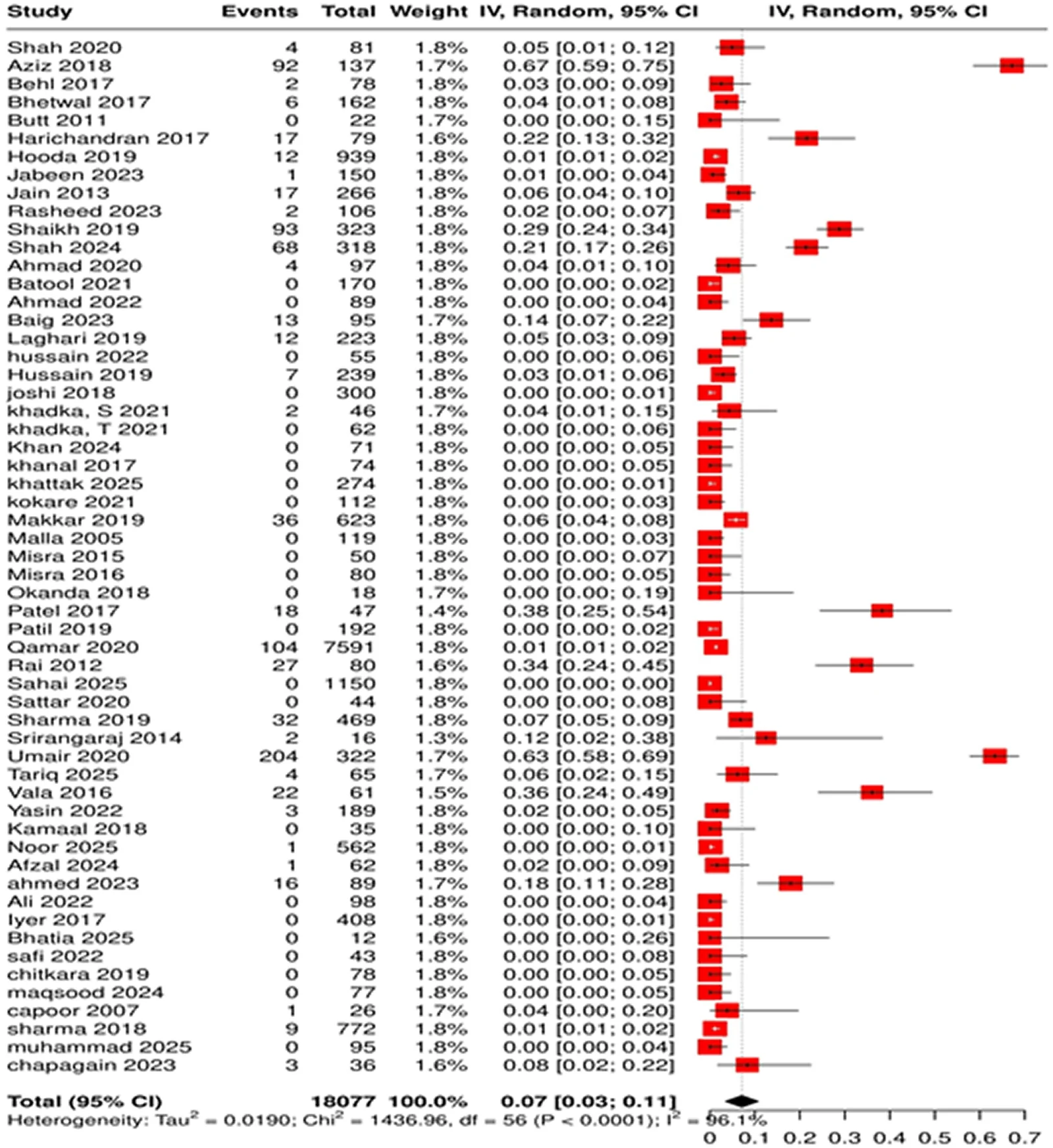
Forest plot of azithromycin resistance in *Salmonella* Typhi.

**Figure 3.**
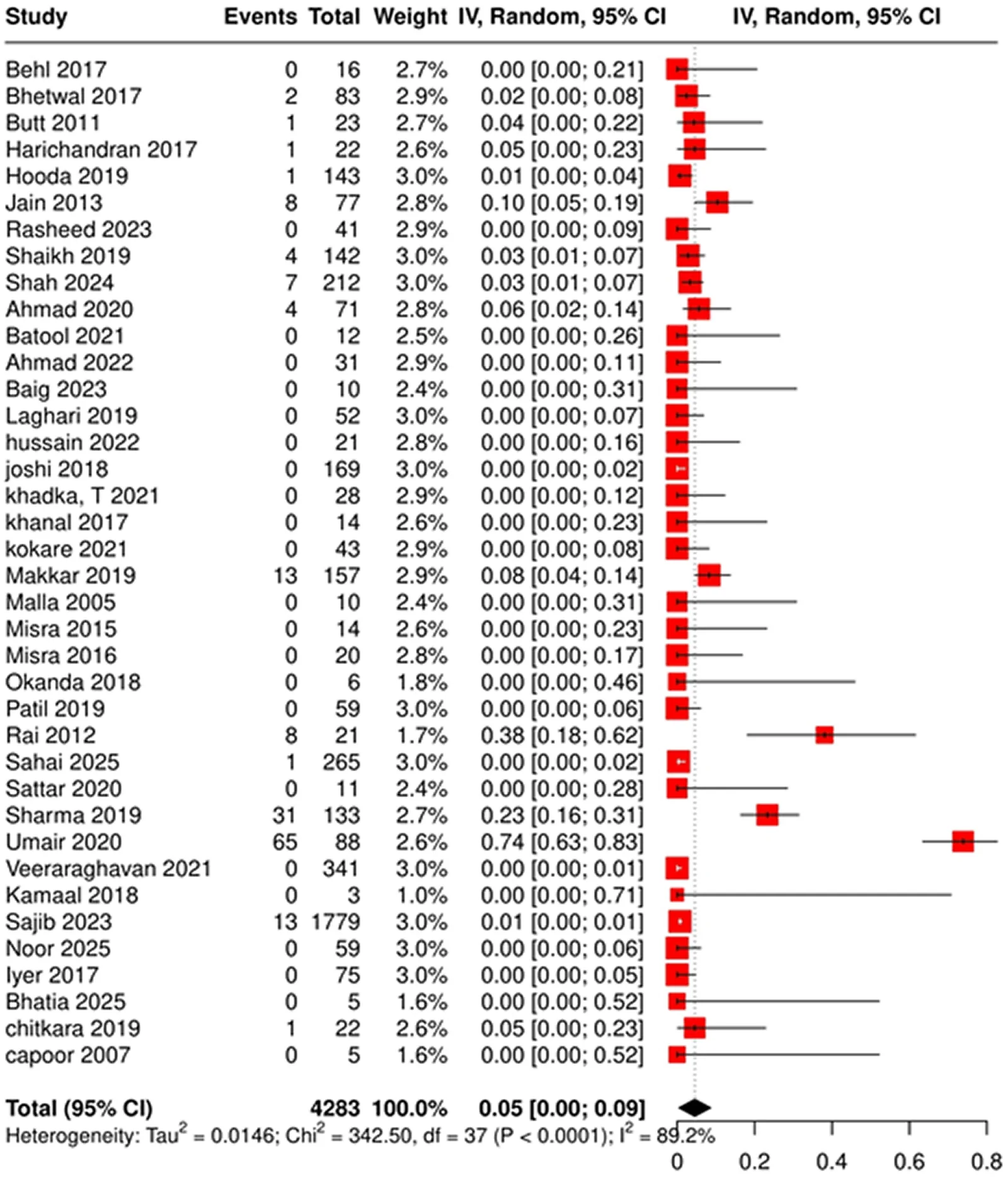
Forest plot of azithromycin resistance in *Salmonella* Paratyphi.

**Figure 4.**
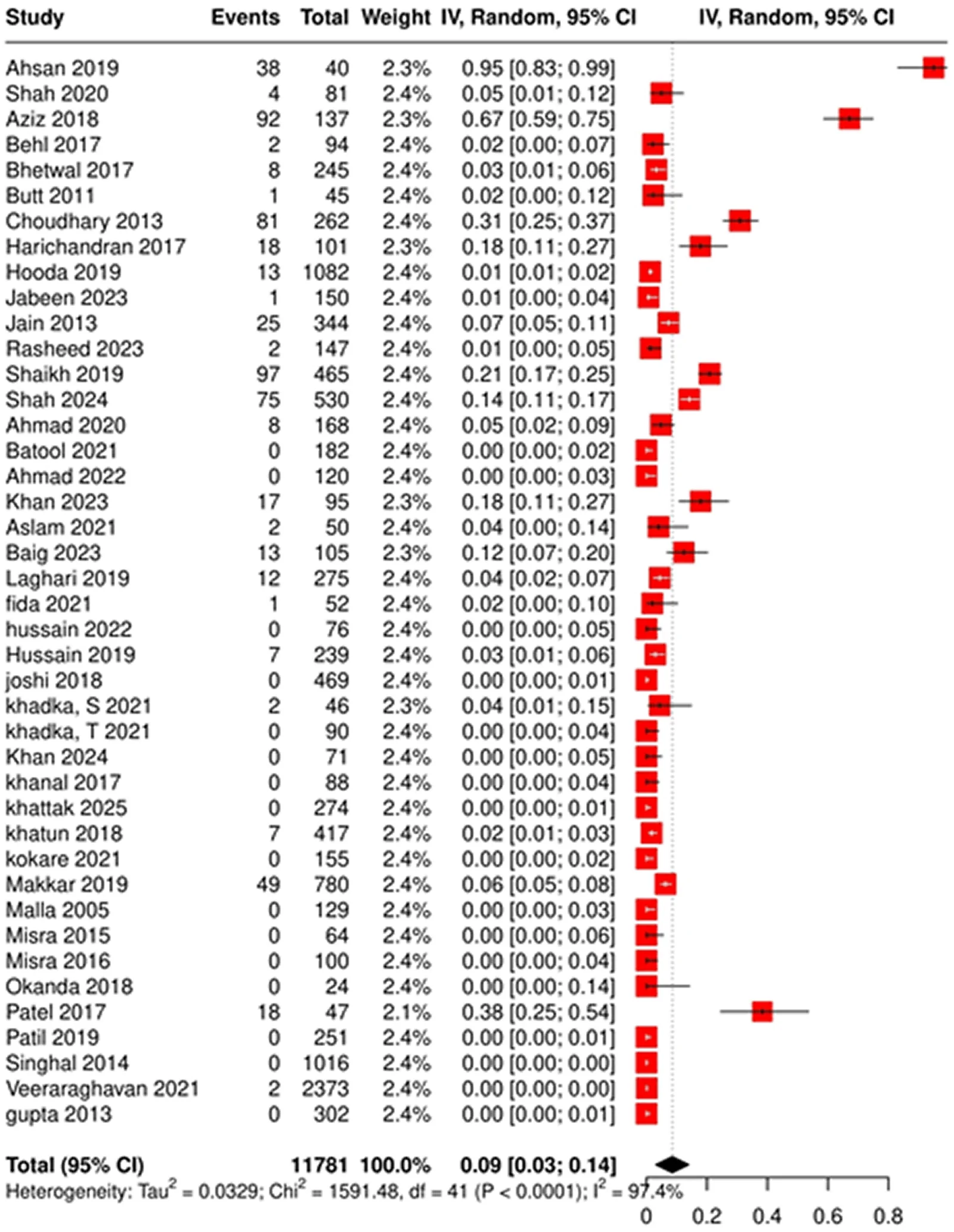
Forest plot of overall azithromycin resistance.

The meta-analysis revealed a pooled azithromycin resistance prevalence of 5% (95% CI: 4%–5%, common-effect; 95% CI: 0%–3%, random-effects) among Salmonella Typhi isolates, based on 14 616 samples, with high heterogeneity observed (*I*^2^ = 97.1%, *P* < .001) (Figure 2). Similarly, for Salmonella Paratyphi, the pooled resistance was 5% (95% CI: 0%–9%; 4283 isolates), also displaying substantial heterogeneity (*I*^2^ = 89.2%, *P* < .001) (Figure 3). When both serotypes were combined, the overall prevalence of azithromycin resistance across all Salmonella isolates was 9% (95% CI: 3%–14%; 11 781 isolates), with similarly high heterogeneity (*I*^2^ = 97.4%, *P* < .001) (Figure 4).

### Sensitivity Analysis

To evaluate whether the introduction of standardized CLSI breakpoints in 2015 influenced the observed prevalence of resistance, we stratified the analysis by study era. For *S. Typhi*, studies conducted before the establishment of CLSI breakpoints (pre-2015; n = 12 studies, 2847 isolates) yielded a pooled resistance prevalence of 4.2% (95% CI: 2.1%–6.3%), compared with 6.1% (95% CI: 4.0%–8.2%) for post-2015 studies (n = 15 studies, 3249 isolates). This difference was not statistically significant (test for subgroup differences: *P* = .21). For *S. Paratyphi*, pre-2015 studies (n = 6 studies, 412 isolates) showed a pooled prevalence of 2.9% (95% CI: 1.0%–4.8%), while post-2015 studies (n = 7 studies, 361 isolates) showed 5.2% (95% CI: 2.1%–8.3%); the difference was not statistically significant (*P* = .34). These findings suggest that the introduction of standardized breakpoints did not substantially alter overall resistance estimates. However, the limited number of pre-2015 studies, particularly for *S. Paratyphi*, constrains the statistical power of this comparison. Full results are presented in Supplementary Table 5.

### Subgroup Analysis by Country

Country-level subgroup analyses were conducted to explore geographical variations in azithromycin resistance. The combined results for *Salmonella* Typhi, *Salmonella* Paratyphi, and the overall pooled prevalence are presented in Table 2.

**Table 2.** Subgroup Analysis of Azithromycin Resistance by Country and Serotype

Country	Serotype	Studies (k)	Isolates (n)	Resistant Isolates (n)	Pooled Prevalence (95% CI)	*I* ^2^ (%)
Pakistan	*S. Typhi*	27	4096	328	8.0 (2.0–15.0)	97.6
Pakistan	*S. Paratyphi*	13	773	54	7.0 (0.0–18.0)	95.1
Pakistan	Combined	19	3262	261	8.0 (1.0–15.0)	96.6
India	*S. Typhi*	21	4934	345	7.0 (2.0–12.0)	91.3
India	*S. Paratyphi*	18	1447	58	4.0 (1.0–7.0)	78.2
India	Combined	14	6358	382	6.0 (1.0–12.0)	94.8
Nepal	*S. Typhi*	6	499	5	1.0 (0.0–3.0)	51.9
Nepal	*S. Paratyphi*	4	135	1	1.0 (0.0–4.0)	0.0
Nepal	Combined	5	598	6	1.0 (0.0–2.0)	56.3
Bangladesh	*S. Typhi*	2	958	10	1.0 (1.0–2.0)	0.0
Bangladesh	*S. Paratyphi*	3	1928	19	1.0 (0.0–1.0)	0.0
Bangladesh	Combined	4	2886	29	1.0 (0.0–2.0)	87.3

For *S. Typhi*, subgroup differences by country were statistically significant (*P* = .037), with Pakistan exhibiting the highest resistance estimate (8%), followed by India (7%), and markedly lower prevalence in Nepal and Bangladesh (1% each), Supplementary Figure 1. In contrast, for *S. Paratyphi*, country-level differences were not significant (*P* = .191), though Pakistan again showed the highest point estimate (7%) (Supplementary Figure 2). When both serotypes were combined, Pakistan demonstrated the highest overall pooled resistance (8%), while Nepal showed the lowest (1%) (Supplementary Figure 3). The test for subgroup differences across the combined estimates remained significant (*P* = .0348). Considerable heterogeneity (*I*^2^ > 75%) was observed in most subgroups, indicating substantial variation in resistance estimates across studies within countries.

### Meta-Regression by Year of Sample Collection

Univariate meta-regression was performed to evaluate potential temporal trends in the logit proportion of azithromycin resistance, using the midpoint year of sample collection for each included study as the predictor variable. Meta-regression for *S. Typhi* indicated no statistically significant temporal trend from 2005 to 2024 (*P* > .05), suggesting that resistance levels have not demonstrated a clear upward or downward trajectory over the study period (Supplementary Figure 4). Similarly, for *S. Paratyphi*, the meta-regression model showed no significant temporal trend (*P* > .05), suggesting a consistent, and relatively low-level resistance profile over time (Supplementary Figure 5). When data for both serotypes were combined, the overall meta-regression confirmed the absence of a significant temporal trend (*P* > .05), reinforcing the conclusion that azithromycin resistance in enteric fever pathogens across South Asia has not fluctuated significantly during the years covered by this analysis (Supplementary Figure 6).

### Publication Bias Assessment

Visual inspection of funnel plots for *S. Typhi*, *S. Paratyphi*, and overall resistance revealed general symmetry around the pooled effect estimate, suggesting a low risk of substantial publication bias. Minor asymmetry observed in some plots is more likely attributable to the high degree of clinical and methodological heterogeneity among included studies, particularly variations in sample size, geographic setting, and laboratory methodologies, rather than systematic nonpublication of negative or null results. This interpretation is supported by the high *I*^2^ values, which quantify substantial heterogeneity, a known contributor to funnel plot asymmetry independent of true publication bias (Supplementary Figure 7).

## DISCUSSION

This systematic review and meta-analysis provide a comprehensive synthesis of available evidence on azithromycin resistance among *Salmonella enterica* serovars *Typhi* and *Paratyphi* in South Asia. Drawing on data from 74 studies conducted over nearly 2 decades and encompassing more than 30 000 isolates, this analysis offers the most extensive regional assessment to date. Overall, we observed a pooled prevalence of 5% for both *S. Typhi* and *S. Paratyphi*, with considerable heterogeneity across studies and countries, and no statistically significant temporal trend detected over the study period.

Several methodological considerations warrant careful interpretation of these findings. CLSI established the first official breakpoints for *S. Typhi* in January 2015 (M100-S25), defining susceptible (MIC ≤16 µg/mL) and resistant (MIC ≥32 µg/mL) categories [85]. Importantly, no CLSI or EUCAST breakpoints exist for *S. Paratyphi* A, B, or C to date [86]. This regulatory gap has necessitated the application of *S. Typhi* interpretive criteria to paratyphoid isolates, despite evidence that the natural MIC distribution of azithromycin for *S. Paratyphi* may differ from that of *S. Typhi* [87]. Our sensitivity analysis comparing pre- and post-2015 studies revealed no statistically significant differences in resistance estimates, suggesting that the introduction of standardized breakpoints did not substantially bias overall prevalence findings. However, the absence of serotype-specific breakpoints for *S. Paratyphi* remains a fundamental limitation that constrains the precision of resistance estimates for this serotype [86].

Additionally, the reliability of azithromycin susceptibility testing methods warrants consideration. Disc diffusion and E-test endpoints can be challenging to interpret due to the trailing growth phenomenon and the relatively narrow zone diameter differences between susceptible and resistant populations [84, 88]. Broth microdilution, the reference method, was employed in a minority of studies, with most relying on disc diffusion or automated systems. Our quality assessment identified 18 studies (24.3%) as low quality due to insufficient reporting of AST methodology, quality control procedures, or external quality assurance participation. The high degree of statistical heterogeneity observed across all analyses (*I*^2^ > 89%) may, in part, reflect these methodological variations and underscores the need for standardized testing protocols in future surveillance efforts [84].

The pooled prevalence of azithromycin resistance in *S. Typhi* was estimated at approximately 5%, with a similar estimate for *S. Paratyphi*. When both serotypes were combined, the overall prevalence increased to 9%, reflecting the cumulative contribution of both pathogens across diverse settings. Although these estimates suggest that azithromycin remains largely effective against enteric fever pathogens in South Asia, the non-negligible proportion of resistant isolates is clinically significant, particularly given the shrinking arsenal of effective oral antibiotics. Even modest increases in resistance prevalence can translate into substantial numbers of treatment failures in regions with high enteric fever incidence.

Country-level subgroup analyses revealed important geographical heterogeneity. Pakistan consistently demonstrated the highest pooled prevalence across *S. Typhi* (8%), *S. Paratyphi* (7%), and combined analyses (8%). This finding is concordant with Pakistan's well-documented burden of MDR and extensively drug-resistant (XDR) *S. Typhi*, extensive empirical antibiotic consumption, and recurrent outbreaks of drug-resistant enteric fever [7, 27, 40].

We hypothesize that several interconnected factors may explain Pakistan's higher resistance prevalence. The country has experienced large-scale outbreaks of XDR *S. Typhi* since 2016, resulting in widespread use of azithromycin as a last-line oral option in outbreak settings [7]. This intensive selective pressure, combined with a high baseline incidence of enteric fever [1, 2], over-the-counter antibiotic availability, and limited antimicrobial stewardship infrastructure, may have created conditions conducive to the emergence and amplification of azithromycin-resistant clones [89]. However, this hypothesis is speculative and would require formal testing through integrated genomic surveillance and prescribing data analysis.

India showed intermediate resistance levels (7% for *S. Typhi*, 4% for *S. Paratyphi*), which may reflect its heterogeneous healthcare landscape, with regional variability in antimicrobial use and resistance patterns [54, 59, 65]. Large multicenter surveillance studies from India contributed substantial weight to the pooled estimates and suggest that, while azithromycin resistance is present, it has not yet reached alarming levels at the national scale [59]. Nevertheless, localized outbreaks or institutional clusters of resistant strains may be obscured in pooled analyses [55], highlighting the importance of ongoing, granular surveillance.

In contrast, Nepal and Bangladesh exhibited substantially lower resistance estimates (∼1% for each serotype), though these findings should be interpreted cautiously due to the smaller number of included studies and isolates from these countries [73, 77, 78, 82]. While the lower prevalence in these settings might reflect differences in antibiotic prescribing practices, healthcare access, or public health infrastructure, the limited data constrain robust comparisons [8, 90]. Notably, no eligible studies were identified from Sri Lanka or Bhutan, despite these countries being included in the search strategy. This represents a concerning gap in the regional surveillance network, particularly given that Sri Lanka has reported rising rates of enteric fever and emerging antimicrobial resistance in recent years [90]. The absence of data from these settings underscores the need for coordinated regional surveillance initiatives to ensure comprehensive geographic coverage.

The observation that subgroup differences were statistically significant for *S. Typhi* but not for *S. Paratyphi* may reflect differences in transmission dynamics, antimicrobial exposure, and biological mechanisms of resistance between the 2 serotypes. *S. Paratyphi* infections are often under-recognized and may be subject to different treatment patterns, potentially influencing resistance selection. However, the smaller number of *S. Paratyphi* isolates across many studies limits the precision of these estimates and warrants further focused investigation.

Meta-regression analyses did not detect a statistically significant association between the year of sample collection and resistance prevalence for either serotype or the combined estimate (*P* > .05 for all analyses). While this absence of a clear trend is reassuring, it should not be interpreted as definitive evidence of stability, given the substantial heterogeneity in study design, geographic coverage, and temporal sampling that may have reduced the sensitivity of our analysis to detect genuine trends [9]. Resistance trajectories may be nonlinear, characterized by sudden increases driven by clonal expansion or changes in treatment guidelines, as previously observed with fluoroquinolone and cephalosporin resistance in Salmonella [5, 6].

Several hypotheses may explain the lack of a detected temporal increase. First, azithromycin may have been used more judiciously in some settings due to antimicrobial stewardship efforts and growing awareness of resistance concerns [89]. Second, the fitness cost associated with certain resistance mechanisms, such as efflux pump overexpression or target site modifications, may limit widespread dissemination in the absence of strong selective pressure [85]. Third, the heterogeneity in study periods and uneven temporal coverage across countries may reduce the sensitivity of meta-regression to detect subtle trends. Continued longitudinal surveillance using standardized methodologies is therefore essential to monitor emerging changes [83].

The clinical significance of in vitro azithromycin resistance warrants consideration. The correlation between azithromycin MIC ≥32 µg/mL and clinical treatment failure has been established for *S. Typhi* [91]. However, the unique pharmacokinetic properties of this macrolide—achieving intracellular concentrations in macrophages and neutrophils 50- to 100-fold higher than serum levels may explain why some patients with resistant isolates are successfully treated [92, 93]. Conversely, treatment failures have been documented even with susceptible isolates, suggesting that factors beyond MIC influence clinical outcomes [86]. The majority of included studies reported only laboratory-based resistance data without linking to patient-level outcomes, precluding systematic analysis of the clinical impact of azithromycin resistance. Our estimates are broadly consistent with, though slightly lower than, findings from previous regional analyses. A 2020 meta-analysis by Browne et al that examined global drug-resistant enteric fever reported azithromycin resistance in *S. Typhi* ranging from 3% to 8% across South Asian countries, with substantial heterogeneity in point estimates [9]. Our pooled estimate of 5% falls within this range and likely reflects the inclusion of more recent studies, including post-2015 data with standardized CLSI breakpoints [85]. In contrast, a 2024 systematic review focusing on Pakistan reported higher estimates (8%–12%) for azithromycin resistance, consistent with our country-specific finding of 8% for Pakistan, though our broader regional estimate is lower [10]. The disparity between regional and Pakistan-specific estimates underscores the importance of geographically stratified analyses and suggests that aggregated regional estimates may underestimate resistance in high-burden settings [9, 10]. For *S. Paratyphi*, our finding of 5% resistance is comparable to the limited estimates available from Southeast Asian surveillance networks, though no comparable meta-analysis exists for direct comparison [83].

The geographical variability in resistance prevalence indicates the need for country-specific and subnational treatment guidelines informed by local resistance data rather than reliance on regional or global averages. Strengthening laboratory capacity for blood culture and AST, particularly in peripheral and rural settings, is critical to support evidence-based prescribing [84]. In addition, integrating azithromycin resistance monitoring into existing enteric fever surveillance platforms can facilitate early detection of emerging resistance patterns [83, 93].

Based on our findings, we recommend 3 priority actions: (1) establishing a regional azithromycin resistance surveillance network with standardized CLSI/EUCAST methodologies and mandatory external quality assurance [84, 85]; (2) developing serotype-specific breakpoints for *S. Paratyphi* to improve resistance classification accuracy [86]; and (3) implementing targeted stewardship interventions in high-prevalence settings, including Pakistan, with monitoring of treatment outcomes to guide empirical therapy decisions [89].

Vaccination and nonpharmacological interventions remain essential components of a comprehensive strategy to control enteric fever and mitigate antimicrobial resistance. The rollout of typhoid conjugate vaccines in several South Asian countries, including Pakistan, offers an opportunity to reduce disease incidence and antibiotic consumption, thereby indirectly slowing the emergence of resistance [94]. Improvements in WASH infrastructure are equally critical to addressing the root causes of transmission [2, 8].

Several limitations should be considered. Substantial heterogeneity was observed across included studies due to differences in study design, population characteristics, geographic settings, sample sizes, and AST methods [9]. Many studies were hospital- or laboratory-based and conducted in urban or tertiary care settings, potentially over-representing severe cases and limiting generalizability to community-level infections [90]. Data from some South Asian countries were sparse or absent, resulting in uneven regional representation. Variations in AST methodologies, interpretive criteria, and the absence of *S. Paratyphi*-specific breakpoints may have led to misclassification of resistance [85, 86]. The observational nature of the included studies and the lack of patient-level data precluded adjustment for important confounders such as prior antibiotic exposure, vaccination status, and clinical outcomes.

Azithromycin resistance among Salmonella Typhi and Paratyphi in South Asia remains present at relatively low prevalence, with no statistically significant temporal trend detected over the study period. However, the substantial heterogeneity in available data and uneven temporal coverage across countries warrant caution in interpreting this as definitive stability. Azithromycin continues to be a valuable therapeutic option for enteric fever; however, the emergence of resistance in a context of limited alternatives highlights the urgent need for sustained surveillance, antimicrobial stewardship, vaccination, and public health interventions. Proactive and coordinated regional efforts are essential to preserve the effectiveness of azithromycin and to prevent further escalation of drug-resistant enteric fever in South Asia.

## CONCLUSION

Azithromycin resistance among *Salmonella* Typhi and Paratyphi in South Asia remains present but relatively low and stable over time, with notable geographical variation and particularly higher prevalence in Pakistan. Azithromycin continues to be a valuable therapeutic option for enteric fever; however, the emergence of resistance in a context of limited alternatives highlights the urgent need for sustained surveillance, antimicrobial stewardship, vaccination, and public health interventions. Proactive and coordinated regional efforts are essential to preserve the effectiveness of azithromycin and to prevent a further escalation of drug-resistant enteric fever in South Asia.

## Supplementary Material

ofag581_Supplementary_Data

## Supplementary Data

Supplementary materials are available at *Open Forum Infectious Diseases* online. Consisting of data provided by the authors to benefit the reader, the posted materials are not copyedited and are the sole responsibility of the authors, so questions or comments should be addressed to the corresponding author.
