# Supplementary material for: Azithromycin Resistance in Salmonella Typhi and Paratyphi Across South Asia: A Systematic Review and Meta-Analysis
Source: Open Forum Infect Dis. 2026 Sep 12;13(9):ofag581. doi: 10.1093/ofid/ofag581 (PMC13598339; doi:10.1093/ofid/ofag581)
Supplement: Supplementary file 1 [file ofag581_supplementary_data.docx]

**SUPPLEMENTARY MATERIALS**

**Figure S1 Subgroup Analysis of S. Typhi Resistance by Country**

**Figure S2 Subgroup Analysis of S. Paratyphi Resistance by Country**

**Figure S3 Subgroup Analysis of Overall Resistance by Country**

**Figure S4:** Meta-Regression of Salmonella Typhi Resistance Over Time

**Figure S5: Meta-Regression of Salmonella Paratyphi Resistance Over Time**

**Figure S6:** **Meta-Regression of Overall Resistance Over Time**

**Figure S7: Funnel Plots for Publication Bias Assessment**

## **Table S1.** PRISMA Checklist

| **Section and Topic** | **Item #** | **Checklist item** | **Location where item is reported** |
| --- | --- | --- | --- |
| **TITLE** | | |  |
| Title | 1 | Identify the report as a systematic review. | 1 |
| **ABSTRACT** | | |  |
| Abstract | 2 | See the PRISMA 2020 for Abstracts checklist. (made as per the Journal guidelines) | 2 |
| **INTRODUCTION** | | |  |
| Rationale | 3 | Describe the rationale for the review in the context of existing knowledge. | 3 |
| Objectives | 4 | Provide an explicit statement of the objective(s) or question(s) the review addresses. | 3 |
| **METHODS** | | |  |
| Eligibility criteria | 5 | Specify the inclusion and exclusion criteria for the review and how studies were grouped for the syntheses. | 4 |
| Information sources | 6 | Specify all databases, registers, websites, organisations, reference lists and other sources searched or consulted to identify studies. Specify the date when each source was last searched or consulted. | 4 |
| Search strategy | 7 | Present the full search strategies for all databases, registers and websites, including any filters and limits used. | Table S3 |
| Selection process | 8 | Specify the methods used to decide whether a study met the inclusion criteria of the review, including how many reviewers screened each record and each report retrieved, whether they worked independently, and if applicable, details of automation tools used in the process. | 4 |
| Data collection process | 9 | Specify the methods used to collect data from reports, including how many reviewers collected data from each report, whether they worked independently, any processes for obtaining or confirming data from study investigators, and if applicable, details of automation tools used in the process. | 4, 5 |
| Data items | 10a | List and define all outcomes for which data were sought. Specify whether all results that were compatible with each outcome domain in each study were sought (e.g., for all measures, time points, analyses), and if not, the methods used to decide which results to collect. | 4, Table 1 |
|  | 10b | List and define all other variables for which data were sought (e.g., participant and intervention characteristics, funding sources). Describe any assumptions made about any missing or unclear information. | 4 |
| Study risk of bias assessment | 11 | Specify the methods used to assess risk of bias in the included studies, including details of the tool(s) used, how many reviewers assessed each study and whether they worked independently, and if applicable, details of automation tools used in the process. | 5, Table S4 |
| Effect measures | 12 | Specify for each outcome the effect measure(s) (e.g. risk ratio, mean difference) used in the synthesis or presentation of results. | 5 |
| Synthesis methods | 13a | Describe the processes used to decide which studies were eligible for each synthesis (e.g. tabulating the study intervention characteristics and comparing against the planned groups for each synthesis (item #5)). | 5, Table 1 |
|  | 13b | Describe any methods required to prepare the data for presentation or synthesis, such as handling of missing summary statistics, or data conversions. | NA |
|  | 13c | Describe any methods used to tabulate or visually display results of individual studies and syntheses. | 4 |
|  | 13d | Describe any methods used to synthesize results and provide a rationale for the choice(s). If meta-analysis was performed, describe the model(s), method(s) to identify the presence and extent of statistical heterogeneity, and software package(s) used. | 5 |
|  | 13e | Describe any methods used to explore possible causes of heterogeneity among study results (e.g. subgroup analysis, meta-regression). | 6 |
|  | 13f | Describe any sensitivity analyses conducted to assess robustness of the synthesized results. | 6 |
| Reporting bias assessment | 14 | Describe any methods used to assess risk of bias due to missing results in a synthesis (arising from reporting biases). | NA |
| Certainty assessment | 15 | Describe any methods used to assess certainty (or confidence) in the body of evidence for an outcome. | NA |
| **RESULTS** | | |  |
| Study selection | 16a | Describe the results of the search and selection process, from the number of records identified in the search to the number of studies included in the review, ideally using a flow diagram. | Figure-1 |
|  | 16b | Cite studies that might appear to meet the inclusion criteria, but which were excluded, and explain why they were excluded. | 6, Table 1 |
| Study characteristics | 17 | Cite each included study and present its characteristics. | Table-1 |
| Risk of bias in studies | 18 | Present assessments of risk of bias for each included study. | Table S4 |
| Results of individual studies | 19 | For all outcomes, present, for each study: (a) summary statistics for each group (where appropriate) and (b) an effect estimate and its precision (e.g. confidence/credible interval), ideally using structured tables or plots. | Table 1, Figure 2,3 |
| Results of syntheses | 20a | For each synthesis, briefly summarise the characteristics and risk of bias among contributing studies. | 5 |
|  | 20b | Present results of all statistical syntheses conducted. If meta-analysis was done, present for each the summary estimate and its precision (e.g. confidence/credible interval) and measures of statistical heterogeneity. If comparing groups, describe the direction of the effect. | 5, 6 Figure 2, 3 |
|  | 20c | Present results of all investigations of possible causes of heterogeneity among study results. | 6 |
|  | 20d | Present results of all sensitivity analyses conducted to assess the robustness of the synthesized results. | NA |
| Reporting biases | 21 | Present assessments of risk of bias due to missing results (arising from reporting biases) for each synthesis assessed. | NA |
| Certainty of evidence | 22 | Present assessments of certainty (or confidence) in the body of evidence for each outcome assessed. | NA |
| **DISCUSSION** | | |  |
| Discussion | 23a | Provide a general interpretation of the results in the context of other evidence. | 6 |
|  | 23b | Discuss any limitations of the evidence included in the review. | 7 |
|  | 23c | Discuss any limitations of the review processes used. | 8 |
|  | 23d | Discuss implications of the results for practice, policy, and future research. | 9 |
| **OTHER INFORMATION** | | |  |
| Registration and protocol | 24a | Provide registration information for the review, including register name and registration number, or state that the review was not registered. | 4 |
|  | 24b | Indicate where the review protocol can be accessed, or state that a protocol was not prepared. | 4 |
|  | 24c | Describe and explain any amendments to information provided at registration or in the protocol. | NA |
| Support | 25 | Describe sources of financial or non-financial support for the review, and the role of the funders or sponsors in the review. | 9 |
| Competing interests | 26 | Declare any competing interests of review authors. | 9 |
| Availability of data, code and other materials | 27 | Report which of the following are publicly available and where they can be found: template data collection forms; data extracted from included studies; data used for all analyses; analytic code; any other materials used in the review. | 9 |

**Table S2. The adjusted search terms as per searched electronic databases 31/12/2025**

| **Database** | **No** | **Search Query** | **Results** | |
| --- | --- | --- | --- | --- |
| **PubMed** | | | |  |
|  | #1 | ("Salmonella typhi" OR "Salmonella paratyphi" OR "S. typhi" OR "S. paratyphi" OR "typhoidal Salmonella" OR "enteric fever") AND ("azithromycin" OR "macrolide") AND ("resistance" OR "susceptibility" OR "antimicrobial resistance" OR "antibiotic resistance") AND ("South Asia" OR "Pakistan" OR "India" OR "Bangladesh" OR "Nepal" OR "Sri Lanka" OR "Bhutan") | 370 | |
| **Science Direct** | | | |  |
|  | #1 | ("Salmonella Typhi" OR "Salmonella Paratyphi" OR "S. Typhi" OR "S. Paratyphi" OR "typhoid" OR "paratyphoid") AND ("azithromycin" OR "macrolide") AND ("resistance" OR "susceptibility" OR "antimicrobial resistance") AND ("South Asia" OR "Pakistan" OR "India" OR "Bangladesh" OR "Nepal" OR "Sri Lanka" OR "Bhutan") | 282 | |
| **Embase** | | | |  |
|  | #1 | ('salmonella typhi'/exp OR 'salmonella paratyphi'/exp OR 'typhoid fever'/exp OR 'paratyphoid fever'/exp) AND ('azithromycin'/exp OR 'macrolide'/exp) AND ('drug resistance'/exp OR 'antibiotic resistance'/exp OR 'susceptibility'/exp) AND ('south asia'/exp OR 'pakistan'/exp OR 'india'/exp OR 'bangladesh'/exp OR 'nepal'/exp OR 'sri lanka'/exp OR 'bhutan'/exp) | 523 | |
| **Web of Science** | | | |  |
|  | #1 | TS=("Salmonella Typhi" OR "Salmonella Paratyphi" OR "S. Typhi" OR "S. Paratyphi" OR "typhoid fever" OR "enteric fever") AND TS=("azithromycin" OR "macrolide") AND TS=("resistance" OR "susceptibility" OR "antimicrobial resistance") AND TS=("South Asia" OR "Pakistan" OR "India" OR "Bangladesh" OR "Nepal" OR "Sri Lanka" OR "Bhutan") | 298 | |
| **Google Scholar** | | | |  |
|  | #1 | ("Salmonella Typhi" OR "Salmonella Paratyphi" OR "typhoid" OR "paratyphoid") AND ("azithromycin" OR "macrolide") AND ("resistance" OR "antimicrobial resistance") AND ("South Asia" OR "Pakistan" OR "India" OR "Bangladesh" OR "Nepal" OR "Sri Lanka" OR "Bhutan") | 380 | |
| **PakMediNet** | | | | |
|  |  | (Salmonella Typhi OR Salmonella Paratyphi OR typhoid OR paratyphoid) AND (azithromycin OR macrolide) AND (resistance OR susceptibility) AND (Pakistan OR South Asia | 10 | |

**Table S3. Inclusion and Exclusion criteria**

**Research Question: What is the prevalence and trend of azithromycin resistance in Salmonella in South asia?**

| **Inclusion** | | **Exclusion** |
| --- | --- | --- |
| **Participants** | Patients diagnosed with Salmonella infection in Pakistan and | None |
| **Intervention** | None |  |
| **Outcome** | Prevalence of resistance to Azitromycin | None |
| **Study Designs** | Observational studies (cross-sectional, cohort, case-control) and surveillance reports | Without clear resistance data, studies were not conducted in Pakistan and were non-peer-reviewed. |
|  | Date of Search- 31 Dec 2025  Published articles and preprint data in the English language | Unavailable full-text articles not in English language |

**Table S4. Quality assessment using a JBI quality assessment tool for prevalence studies.**

**JBI Checklist Criteria for Prevalence Studies:**

| **Criterion** | **Description** |
| --- | --- |
| **D1** | Was the sample frame appropriate to address the target population? |
| **D2** | Were study participants sampled in an appropriate way? |
| **D3** | Was the sample size adequate? |
| **D4** | Were the study subjects and the setting described in detail? |
| **D5** | Was the data analysis conducted with sufficient coverage of the identified sample? |
| **D6** | Were valid methods used for the identification of the condition (i.e., antimicrobial susceptibility testing)? |
| **D7** | Was the condition measured in a standard, reliable way for all participants? |
| **D8** | Was there appropriate statistical analysis? |
| **D9** | Was the response rate adequate? |

| **Study ID** | **D1** | **D2** | **D3** | **D4** | **D5** | **D6** | **D7** | **D8** | **D9** | **Overall Quality** |
| --- | --- | --- | --- | --- | --- | --- | --- | --- | --- | --- |
| Ahsan 2019 | Y | U | U | Y | U | Y | Y | U | U | Moderate |
| Shah 2020 | Y | U | U | Y | U | Y | Y | U | U | Moderate |
| Aziz 2018 | Y | U | U | Y | U | Y | Y | U | U | Moderate |
| Behl 2017 | Y | U | U | Y | U | Y | Y | U | U | Moderate |
| Bhetwal 2017 | Y | U | U | Y | U | Y | Y | U | U | Moderate |
| Butt 2011 | Y | U | U | Y | U | Y | Y | U | U | Moderate |
| Choudhary 2013 | Y | U | U | Y | U | Y | Y | U | U | Moderate |
| Harichandran 2017 | Y | U | U | Y | U | Y | Y | U | U | Moderate |
| Hooda 2019 | Y | U | U | Y | U | Y | Y | U | U | Moderate |
| Jabeen 2023 | Y | U | U | Y | U | Y | Y | U | U | Moderate |
| Jain 2013 | Y | U | U | Y | U | Y | Y | U | U | Moderate |
| Rasheed 2023 | Y | U | U | Y | U | Y | Y | U | U | Moderate |
| Shaikh 2019 | Y | U | U | Y | U | Y | Y | U | U | Moderate |
| Shah 2024 | Y | U | U | Y | U | Y | Y | U | U | Moderate |
| Ahmad 2020 | Y | U | U | Y | U | Y | Y | U | U | Moderate |
| Batool 2021 | Y | U | U | Y | U | Y | Y | U | U | Moderate |
| Ahmad 2022 | Y | U | U | Y | U | Y | Y | U | U | Moderate |
| Khan 2023 | Y | U | U | Y | U | Y | Y | U | U | Moderate |
| Aslam 2021 | Y | U | U | Y | U | Y | Y | U | U | Moderate |
| Baig 2023 | Y | U | U | Y | U | Y | Y | U | U | Moderate |
| Laghari 2019 | Y | U | U | Y | U | Y | Y | U | U | Moderate |
| Fida 2021 | Y | U | U | Y | U | U | U | U | U | Low |
| Hussain 2022 | Y | U | U | Y | U | Y | Y | U | U | Moderate |
| Hussain 2019 | Y | U | U | Y | U | Y | Y | U | U | Moderate |
| Joshi 2018 (India) | Y | U | U | Y | U | Y | Y | U | U | Moderate |
| Khadka, S 2021 | Y | U | U | Y | U | Y | Y | U | U | Moderate |
| Khadka, T 2021 | Y | U | U | Y | U | Y | Y | U | U | Moderate |
| Khan 2024 | Y | U | U | Y | U | Y | Y | U | U | Moderate |
| Khanal 2017 | Y | U | U | Y | U | Y | Y | U | U | Moderate |
| Khattak 2025 | Y | U | U | Y | U | Y | Y | U | U | Moderate |
| Khatun 2018 | Y | U | U | Y | U | Y | Y | U | U | Moderate |
| Kokare 2021 | Y | U | U | Y | U | Y | Y | U | U | Moderate |
| Makkar 2019 | Y | U | U | Y | U | Y | Y | U | U | Moderate |
| Malla 2005 | Y | U | U | Y | U | Y | Y | U | U | Moderate |
| Misra 2015 | Y | U | U | Y | U | Y | Y | U | U | Moderate |
| Misra 2016 | Y | U | U | Y | U | Y | Y | U | U | Moderate |
| Okanda 2018 | Y | U | U | Y | U | Y | Y | U | U | Moderate |
| Patel 2017 | Y | U | U | Y | U | Y | Y | U | U | Moderate |
| Patil 2019 | Y | U | U | Y | U | Y | Y | U | U | Moderate |
| Qamar 2020 | Y | U | U | Y | U | Y | Y | U | U | Moderate |
| Qamar 2025 | Y | U | U | Y | U | U | U | U | U | Low |
| Rai 2012 | U | U | U | Y | U | Y | Y | U | U | Moderate |
| Sahai 2025 | U | U | U | Y | U | Y | Y | U | U | Moderate |
| Sattar 2020 | U | U | U | Y | U | Y | Y | U | U | Moderate |
| Sharma 2019 | Y | U | U | Y | U | Y | Y | U | U | Moderate |
| Singhal 2014 | Y | U | U | Y | U | Y | Y | U | U | Moderate |
| Srirangaraj 2014 | Y | U | U | Y | U | Y | Y | U | U | Moderate |
| Umair 2020 | Y | U | U | Y | U | U | U | U | U | Low |
| Tariq 2025 | Y | U | U | Y | U | Y | Y | U | U | Moderate |
| Vala 2016 | Y | U | U | Y | U | Y | Y | U | U | Moderate |
| Veeraraghavan 2021 | Y | U | U | Y | U | Y | Y | U | U | Moderate |
| Yasin 2022 | Y | U | U | Y | U | U | U | U | U | Low |
| Kamaal 2018 | Y | U | U | Y | U | Y | Y | U | U | Moderate |
| Sajib 2023 | Y | U | U | Y | U | Y | Y | U | U | Moderate |
| Noor 2025 | Y | U | U | Y | U | Y | Y | U | U | Moderate |
| Afzal 2024 | Y | U | U | Y | U | Y | Y | U | U | Moderate |
| Ahmed 2023 | Y | U | U | Y | U | Y | Y | U | U | Moderate |
| Ali 2022 | Y | U | U | Y | U | Y | Y | U | U | Moderate |
| Iyer 2017 | Y | U | U | Y | U | Y | Y | U | U | Moderate |
| Bhatia 2025 | Y | U | U | Y | U | Y | Y | U | U | Moderate |
| Safi 2022 | Y | U | U | Y | U | Y | Y | U | U | Moderate |
| Mahmood 2024 | Y | U | U | Y | U | Y | Y | U | U | Moderate |
| Chitkara 2019 | Y | U | U | Y | U | Y | Y | U | U | Moderate |
| Hayat 2025 | Y | U | U | Y | U | Y | Y | U | U | Moderate |
| Gupta 2013 | Y | U | U | Y | U | Y | Y | U | U | Moderate |
| Maqsood 2024 | Y | U | U | Y | U | Y | Y | U | U | Moderate |
| Capoor 2007 | Y | U | U | Y | U | Y | Y | U | U | Moderate |
| Sharma 2018 | Y | U | U | Y | U | Y | Y | U | U | Moderate |
| Joshi 2018 (Nepal) | Y | U | U | Y | U | Y | Y | U | U | Moderate |
| Muhammad 2025 | Y | U | U | Y | U | Y | Y | U | U | Moderate |
| Chapagain 2023 | Y | U | U | Y | U | U | U | U | U | Low |
| Garg 2013 | Y | U | U | Y | U | Y | Y | U | U | Moderate |
| Qaiser 2011 | Y | U | U | Y | U | Y | Y | U | U | Moderate |
| Ahmad 2023 (Lahore) | Y | U | U | Y | U | Y | Y | U | U | Moderate |

- **Y** = Yes
- **N** = No
- **U** = Unclear
- **Overall Quality**:
  - **Moderate**: Basic study details (D4) and valid AST methods (D6, D7) reported.
  - **Low**: Key methodological details (e.g., AST method) not reported (D6, D7 unclear)
- **Table S5: Sensitivity Analysis of Azithromycin Resistance by Breakpoint Era (Pre-2015 vs. Post-2015)**

| Serotype | Breakpoint Era | Studies (k) | Isolates (n) | Resistant (n) | Pooled Prevalence (95% CI) | I² (%) | P-value* |
| --- | --- | --- | --- | --- | --- | --- | --- |
| *S. Typhi* | Pre-2015 (no CLSI breakpoints) | 12 | 2,847 | 114 | 4.2 (2.1-6.3) | 86.2 | — |
| *S. Typhi* | Post-2015 (CLSI breakpoints available) | 15 | 3,249 | 195 | 6.1 (4.0-8.2) | 91.7 | 0.21 |
| *S. Paratyphi* | Pre-2015 (no CLSI breakpoints) | 6 | 412 | 12 | 2.9 (1.0-4.8) | 72.4 | — |
| *S. Paratyphi* | Post-2015 (CLSI breakpoints available) | 7 | 361 | 18 | 5.2 (2.1-8.3) | 81.6 | 0.34 |

References

1.	Rahman, S.I.A., et al., *Comparative Epidemiology of Salmonella enterica Serovers Paratyphi A and Typhi Causing Enteric Fever, Bangladesh, 2018–2020.* Emerging Infectious Diseases, 2025. **31**(10): p. 1922.

2.	Piovani, D., et al., *The global burden of enteric fever, 2017–2021: a systematic analysis from the global burden of disease study 2021.* EClinicalMedicine, 2024. **77**.

3.	Hamdulay, K., et al., *Evolving epidemiology and antibiotic resistance in enteric fever: a comprehensive review.* Cureus, 2024. **16**(6).

4.	Gupta, P.K. and J. Gupta, *Former first-line antibiotics for treatment of enteric fever.* The Lancet Infectious Diseases, 2016. **16**(8): p. 892-893.

5.	Rowe, B., L.R. Ward, and E.J. Threlfall, *Multidrug-resistant Salmonella typhi: a worldwide epidemic.* Clinical infectious diseases, 1997. **24**(Supplement_1): p. S106-S109.

6.	Dalhoff, A., *Global fluoroquinolone resistance epidemiology and implictions for clinical use.* Interdisciplinary perspectives on infectious diseases, 2012. **2012**(1): p. 976273.

7.	Bakhat, S., et al., *EXTENSIVELY DRUG RESISTANT (XDR) SALMONELLA TYPHI RED FLAG FOR CLINICIANS AT KARACHI.* Infectious Diseases Journal of Pakistan, 2023. **32**(4): p. 138-142.

8.	Chereau, F., et al., *Risk assessment for antibiotic resistance in South East Asia.* Bmj, 2017. **358**.

9.	Browne, A.J., et al., *Drug-resistant enteric fever worldwide, 1990 to 2018: a systematic review and meta-analysis.* BMC medicine, 2020. **18**(1): p. 1.

10.	Ullah, A., et al. *Prevalence of the Antibiotic Resistance of Salmonella typhi and Salmonella paratyphi in Pakistan: A Systematic Review and Meta-analysis*. in *Open Forum Infectious Diseases*. 2025. Oxford University Press US.

11.	Ali Shah, S.A., et al., *Antimicrobial Sensitivity Pattern of Salmonella Typhi: Emergence of Resistant Strains.* Cureus, 2020. **12**(11): p. e11778.

12.	Aziz, S. and L. Malik, *Emergence of Multi-Resistant Enteric Infection In A Paediatric Unit Of Karachi, Pakistan.* J Pak Med Assoc, 2018. **68**(12): p. 1848-1850.

13.	Butt, F. and F. Sultan, *In vitro activity of azithromycin in Salmonella isolates from Pakistan.* J Infect Dev Ctries, 2011. **5**(5): p. 391-5.

14.	Jabeen, K., et al., *Reporting of Azithromycin Activity against Clinical Isolates of Extensively Drug-Resistant Salmonella enterica Serovar Typhi.* Am J Trop Med Hyg, 2023. **108**(5): p. 942-947.

15.	Rasheed, F., et al., *Trend of antimicrobial resistance among Salmonella typhi isolated from the pediatric population presenting at a tertiary care hospital in Lahore.* Biomedica, 2023. **39**(2): p. 61-66.

16.	Shaikh, A.A., A. Shaikh, and A. Tahir, *Antimicrobial resistance trends of typhoidal salmonellae in southern Pakistan.* Rawal Medical Journal, 2019. **44**(1): p. 7-7.

17.	Shah, M.W., I. Ali, and R. Zaman, *Antimicrobial susceptibility patterns of Salmonella typhi and Salmonella paratyphi in tertiary care hospitals.* Pakistan Journal of Medical & Health Sciences, 2024. **18**(01): p. 59-59.

18.	Ahmad, S., et al., *Trends in antibiotic susceptibility of enteric fever isolates among children attending a tertiary care hospital of Peshawar, KP.* Journal of Rehman Medical Institute, 2020. **6**(3): p. 20-23.

19.	Batool, A., et al., *Prevalence of multi-drug resistant and extensively drug-resistant Salmonella enterica serovar Typhi recovered from pediatrics' septicemia patients in Lahore.* Pak J Med Health Sci, 2021. **15**(4): p. 843-5.

20.	Khan, H.I., et al., *Antibiotic sensitivity pattern in blood culture positive typhoid fever.* Methods, 2020.

21.	Khan, A.M., et al., *Typhoid-related positive pattern of antibiotic sensitivity in blood cultures A cross-sectional study.* Journal of Bacha Khan Medical College, 2023. **4**(02): p. No. 14-17.

22.	Aslam, A., et al., *Trends of antimicrobial resistance in typhoidal strains of salmonella in a tertiary care hospital in Pakistan.* Cureus, 2021. **13**(1).

23.	Baig, U., S. Muslim Mehdi, and N. Iftikhar, *A pattern of antibiotic drug resistance of Salmonella Typhi and Salmonella Paratyphi among children with enteric fever in a tertiary care hospital in Lahore, Pakistan.* Croatian Medical Journal, 2023. **64**(4): p. 256-264.

24.	Laghari, G.S., et al., *Antimicrobial susceptibility patterns of Salmonella species in Southern Pakistan.* Cureus, 2019. **11**(4).

25.	Fida, S., et al., *Clinical Perspectives of Multiple and Extensively Drug-Resistant Typhoid; result from a tertiary care hospital from Pakistan.* The Journal of Infection in Developing Countries, 2021. **15**(04): p. 530-537.

26.	Hussain, M.A., et al., *Extensively drug-resistant typhoidal salmonellae: are these bugs swarming into suburban and rural areas of Pakistan?* Cureus, 2022. **14**(6).

27.	Hussain, A., et al., *Typhoidal Salmonella strains in Pakistan: an impending threat of extensively drug-resistant Salmonella Typhi.* European Journal of Clinical Microbiology & Infectious Diseases, 2019. **38**(11): p. 2145-2149.

28.	Khan, M., et al., *A comparable risk of extensively drug-resistant typhoid fever in the pediatric cohort during the COVID-19 pandemic.* International journal of health sciences, 2024. **18**(1): p. 24.

29.	Khattak, Z., et al., *Prevalence and antimicrobial susceptibility of Salmonella enterica Typhi in febrile patients: a cross-sectional study.* The Journal of Infection in Developing Countries, 2025. **19**(06): p. 904-912.

30.	Hayat, B., et al., *The characteristic of antibiotic drug resistance of Salmonella Typhi isolated from tertiary care hospital in Faisalabad.* Pak J Pharm Sci, 2025. **38**(5): p. 1875-1882.

31.	Sattar, A., et al., *Current trends in antimicrobial susceptibility pattern of Salmonella typhi and paratyphi.* Rawal Medical Journal, 2020. **45**(2): p. 291-4.

32.	Umair, M. and S.A. Siddiqui, *Antibiotic susceptibility patterns of Salmonella typhi and Salmonella paratyphi in a tertiary care hospital in Islamabad.* Cureus, 2020. **12**(9).

33.	Tariq, H., et al., *Molecular characterization of extensively drug-resistant Salmonella serovar Typhi in patients with gastrointestinal complications in Quetta, Pakistan.* Pakistan Journal of Pharmaceutical Sciences, 2025. **38**(4).

34.	Yasin, N., et al., *Salmonella typhi from Northwest Pakistan: Molecular strain typing and drug resistance signature.* Microbial Drug Resistance, 2022. **28**(1): p. 120-126.

35.	Noor B, U.K., Suliman M, , *Emerging trends of antimicrobial resistance in Salmonella Typhi and Paratyphi A: a three-year surveillance report from Khyber Pakhtunkhwa, Pakistan. .* medtigo J Med, 2025. **3**(4).

36.	Dr. Tehmina Afzal, D.M.S.R., Dr. Shafaq Aijaz, Dr. Qurat ul Ain, & Dr. Ali Kashif Malik., *EMERGING TRENDS IN ANTIMICROBIAL RESISTANCE OF SALMONELLA TYPHI: A STUDY FROM TERTIARY CARE HOSPITAL, NAWABSHAH PAKISTAN. .* Journal of Population Therapeutics and Clinical Pharmacology,, 2024. **31**(7): p. 1659-1667.

37.	Ahmad, S., et al., *Current Antibiotics Resistance Patterns of Salmonella typhi in District Peshawar, Khyber Pakhtunkhwa, Pakistan.* Current Trends in OMICS, 2023. **2**(2): p. 67-78.

38.	Ali, A., et al., *Frequency and antimicrobial resistance profile of Salmonella typhi isolated from district buner.* Journal of Medical Sciences, 2022. **30**(3): p. 185-189.

39.	Safi, A.U.R., et al., *Molecular identification and differential proteomics of drug resistant Salmonella Typhi.* Diagnostic Microbiology and Infectious Disease, 2023. **105**(4): p. 115883.

40.	Mahmood, A., et al., *Extensively drug-resistant Salmonella typhi infection in adults; experience from a tertiary care hospital.* Pakistan Journal of Medical Sciences, 2024. **40**(6): p. 1219.

41.	Tariq, H., et al., *Molecular characterization of extensively drug-resistant Salmonella serovar Typhi in patients with gastrointestinal complications in Quetta, Pakistan.* Pak J Pharm Sci, 2025. **38**(4): p. 1354-1367.

42.	Maqsood, S., et al., *Synergistic effects of silver nanoparticles in combination with ineffective antibiotics against multidrug resistant Salmonella typhi.* Pak J Med Sci, 2024. **40**(6): p. 1168-1173.

43.	Qaiser, S., et al., *In vitro susceptibility of typhoidal salmonellae against newer antimicrobial agents: a search for alternate treatment options.* Journal of the Pakistan Medical Association, 2011. **61**(5): p. 462.

44.	Muhammad, et al., *Comprehensive Molecular Profiling of AcrAB-TolC Efflux Pump Genes in Salmonella typhi Isolates from Typhoid Infected Patients.* Current Microbiology, 2025. **82**(10): p. 470.

45.	Ahmad, M., N. Shah, and M.A. Siddiqui, *Frequency and Antibiotics sensitivity pattern of culture-positive Salmonella Typhi in Children.* J Coll Physicians Surg Pak, 2023. **33**(03): p. 303-307.

46.	Behl, P., et al., *Patterns in antimicrobial susceptibility of Salmonellae isolated at a tertiary care hospital in northern India.* Indian Journal of Medical Research, 2017. **145**(1): p. 124-128.

47.	Choudhary, A., et al., *Antimicrobial susceptibility of Salmonella enterica serovars in a tertiary care hospital in southern India.* Indian Journal of medical research, 2013. **137**(4): p. 800-802.

48.	Harichandran, D. and K.R. Dinesh, *Antimicrobial susceptibility profile, treatment outcome and serotype distribution of clinical isolates of Salmonella enterica subspecies enterica: a 2-year study from Kerala, South India.* Infect Drug Resist, 2017. **10**: p. 97-101.

49.	Jain, S. and T.D. Chugh, *Antimicrobial resistance among blood culture isolates of Salmonella enterica in New Delhi.* The Journal of Infection in Developing Countries, 2013. **7**(11): p. 788-795.

50.	Joshi, R.D., et al., *Antimicrobial sensitivity trend in blood culture positive enteric fever.* Journal of Nepal Health Research Council, 2018. **16**(02): p. 228-232.

51.	Kokare, R., et al., *Minimum inhibitory concentration (MIC) of Ceftriaxone and Azithromycin for blood culture isolates of Salmonella enterica spp.* The Journal of Infection in Developing Countries, 2021. **15**(4): p. 538-543.

52.	Makkar, A., et al., *Epidemiological profile and antimicrobial resistance pattern of enteric fever in a tertiary care hospital of North India–a seven year ambispective study.* Acta Medica, 2019. **61**(4): p. 125-130.

53.	PatEl, S.R., et al., *Drug resistance pattern in the recent isolates of Salmonella Typhi with special reference to cephalosporins and azithromycin in the Gangetic plain.* Journal of clinical and diagnostic research: JCDR, 2017. **11**(6): p. DM01.

54.	Rai, S., et al., *Rationale of azithromycin prescribing practices for enteric fever in India.* Indian Journal of Medical Microbiology, 2012. **30**(1): p. 30-33.

55.	Sahai, N., et al., *Antimicrobial susceptibility trends of S. Typhi and S. Paratyphi in a post-COVID-19 pandemic India, from a multicenter surveillance network.* Sci Rep, 2025. **15**(1): p. 13777.

56.	Sharma, P., et al., *Azithromycin resistance mechanisms in typhoidal salmonellae in India: A 25 years analysis.* Indian J Med Res, 2019. **149**(3): p. 404-411.

57.	Singhal, L., et al., *Trends in antimicrobial susceptibility of Salmonella Typhi from North India (2001-2012).* Indian J Med Microbiol, 2014. **32**(2): p. 149-52.

58.	Srirangaraj, S., A. Kali, and M.V. Charles, *A study of antibiogram of Salmonella enterica serovar Typhi isolates from Pondicherry, India.* Australas Med J, 2014. **7**(4): p. 185-90.

59.	Vala, S., et al., *Resistance Patterns of Typhoid Fever in Children: A Longitudinal Community-Based Study.* Am J Ther, 2016. **23**(5): p. e1151-4.

60.	Veeraraghavan, B., et al., *Evaluation of antimicrobial susceptibility profile in Salmonella typhi and Salmonella paratyphi A: presenting the current scenario in India and strategy for future management.* The Journal of Infectious Diseases, 2021. **224**(Supplement_5): p. S502-S516.

61.	Kamaal, C.M., et al., *Clinical profile and drug resistance pattern of typhoid fever in children: a prospective hospital-based study.* Asian Pacific J Health Sci, 2018. **5**: p. 210-16.

62.	Iyer, R., et al., *Prevalence and trends in the antimicrobial susceptibility pattern of Salmonella enterica serovars Typhi and Paratyphi A among children in a pediatric tertiary care hospital in South India over a period of ten years: a retrospective study.* European Journal of Clinical Microbiology & Infectious Diseases, 2017. **36**(12): p. 2399-2404.

63.	Bhatia, M., et al., *Genomic characterization and antimicrobial resistance profile of Salmonella spp. clinical isolates: A single-centre hospital-based exploratory study from Uttarakhand, India.* Microbial Pathogenesis, 2025: p. 107686.

64.	Chitkara, A.J., et al., *Clinico-bacteriological profile of typhoid fever in a private sector hospital in New Delhi.* Indian Pediatrics, 2019. **56**(12): p. 1033-1036.

65.	Capoor, M.R., et al., *In vitro activity of azithromycin, newer quinolones and cephalosporins in ciprofloxacin-resistant Salmonella causing enteric fever.* Journal of Medical Microbiology, 2007. **56**(11): p. 1490-1494.

66.	Sharma, P., et al., *Changing trends of culture-positive typhoid fever and antimicrobial susceptibility in a tertiary care North Indian Hospital over the last decade.* Indian J Med Microbiol, 2018. **36**(1): p. 70-76.

67.	Misra, R., et al., *Absence of multidrug resistance in Salmonella enterica serotypes Typhi and Paratyphi A isolates with intermediate susceptibility to ciprofloxacin.* Transactions of The Royal Society of Tropical Medicine and Hygiene, 2015. **109**(8): p. 538-540.

68.	Misra, R. and K.N. Prasad, *Antimicrobial susceptibility to azithromycin among Salmonella enterica Typhi and Paratyphi A isolates from India.* Journal of medical microbiology, 2016. **65**(12): p. 1536-1539.

69.	Patil, N. and P. Mule, *Sensitivity pattern of Salmonella typhi and paratyphi A isolates to chloramphenicol and other anti-typhoid drugs: an in vitro study.* Infection and Drug Resistance, 2019: p. 3217-3225.

70.	Gupta, V., et al., *Trends in the antibiotic resistance patterns of enteric fever isolates–a three year report from a tertiary care centre.* The Malaysian journal of medical sciences: MJMS, 2013. **20**(4): p. 71.

71.	Garg, A., et al., *Antimicrobial resistance pattern and in vivo activity of azithromycin in Salmonella isolates.* Indian Journal of Medical Microbiology, 2013. **31**(3): p. 287-289.

72.	Bhetwal, A., et al., *Enteric fever caused by Salmonella enterica serovars with reduced susceptibility of fluoroquinolones at a community based teaching hospital of Nepal.* International journal of microbiology, 2017. **2017**(1): p. 2869458.

73.	Khadka, P., J. Thapaliya, and S. Thapa, *Susceptibility pattern of Salmonella enterica against commonly prescribed antibiotics, to febrile-pediatric cases, in low-income countries.* BMC pediatrics, 2021. **21**(1): p. 38.

74.	Khadka, S., et al., *Antimicrobial resistance in Salmonella Typhi isolated from a referral Hospital of Kathmandu, Nepal.* Microbiology insights, 2021. **14**: p. 11786361211056350.

75.	Khanal, P.R., et al., *Renaissance of Conventional First‐Line Antibiotics in Salmonella enterica Clinical Isolates: Assessment of MICs for Therapeutic Antimicrobials in Enteric Fever Cases from Nepal.* BioMed Research International, 2017. **2017**(1): p. 2868143.

76.	Kansakar, P., et al., *Epidemiology of typhoid and paratyphoid fever in Kathmandu: two years study and trends of antimicrobial resistance.* Journal of the Nepal Medical Association, 2005. **44**(157).

77.	Shah, G., A. Ghimire, and S. Shrestha, *Clinical profile of culture proven enteric fever in children at university teaching hospital, Nepal.* Journal of Patan Academy of Health Sciences, 2014. **1**(2): p. 42-45.

78.	Chapagain, R.H., et al., *Burden of enteric fever and antibiotic sensitivity in Nepalese Children Prior to Typhoid Vaccine in National Immunization Program.* J Nepal Health Res Counc, 2023. **21**(2): p. 297-302.

79.	Ahsan, S. and S. Rahman, *Azithromycin resistance in clinical isolates of Salmonella enterica serovars Typhi and Paratyphi in Bangladesh.* Microbial Drug Resistance, 2019. **25**(1): p. 8-13.

80.	Hooda, Y., et al., *Molecular mechanism of azithromycin resistance among typhoidal Salmonella strains in Bangladesh identified through passive pediatric surveillance.* PLoS neglected tropical diseases, 2019. **13**(11): p. e0007868.

81.	Khatun, H., et al., *Clinical profile, antibiotic susceptibility pattern of bacterial isolates and factors associated with complications in culture‐proven typhoid patients admitted to an urban hospital in Bangladesh.* Tropical medicine & international health, 2018. **23**(4): p. 359-366.

82.	Okanda, T., et al., *Characteristics of resistance mechanisms and molecular epidemiology of fluoroquinolone-nonsusceptible Salmonella enterica serovar Typhi and Paratyphi A isolates from a Tertiary Hospital in Dhaka, Bangladesh.* Microbial Drug Resistance, 2018. **24**(10): p. 1460-1465.

83.	Sajib, M.S., et al., *Trends in antimicrobial resistance amongst Salmonella Paratyphi A isolates in Bangladesh: 1999–2021.* PLOS Neglected Tropical Diseases, 2023. **17**(11): p. e0011723.

84.	Qamar, F.N., et al., *Antimicrobial resistance in typhoidal Salmonella: surveillance for enteric fever in Asia project, 2016–2019.* Clinical Infectious Diseases, 2020. **71**(Supplement_3): p. S276-S284.

85.	Rahman, M.M., et al., *An overview of antimicrobial stewardship optimization: the use of antibiotics in humans and animals to prevent resistance.* Antibiotics, 2022. **11**(5): p. 667.

86.	Andersson, D.I. and D. Hughes, *Antibiotic resistance and its cost: is it possible to reverse resistance?* Nature Reviews Microbiology, 2010. **8**(4): p. 260-271.

87.	Zhu, Z., X. Hua, and Y. Ke, *Azithromycin resistance in non-typhoidal Salmonella: a review.* Journal of Antimicrobial Chemotherapy, 2025: p. dkaf341.

88.	Hooda, Y., et al., *Mass azithromycin administration: considerations in an increasingly resistant world.* BMJ Global Health, 2020. **5**(6).

89.	Carter, A.S., S.P. Luby, and D.O. Garrett, *Introducing typhoid conjugate vaccine in South Asia: lessons from the surveillance for enteric fever in Asia project.* Clinical Infectious Diseases, 2020. **71**(Supplement_3): p. S191-S195.

90.	Ahmed, S., et al., *Trends in antibiotic susceptibility of enteric fever isolates among children attending a tertiary care hospital of Peshawar, KP.* Journal of Rehman Medical Institute, 2020. **6**(3): p. 20-23.
